# Strain-Specific Fungal–Bacterial Co-Inoculation Regulates Rhizosphere Microecology and Plant–Soil–Microbiome Responses in Conifer Seedlings

**DOI:** 10.3390/microorganisms14071436

**Published:** 2026-06-30

**Authors:** Qian Song, Xiaoshuang Song, Xun Deng, Jian Liang

**Affiliations:** 1 State Key Laboratory of Plateau Ecology and Agriculture, Qinghai University, Xining 810016, China; 2023990003@qhu.edu.cn; 2 Institute of Forestry Protection, Heilongjiang Forestry Academy, Harbin 150040, China; sxshappy1977@163.com

**Keywords:** fungal–bacterial co-inoculation, keystone taxa, plant growth-promoting rhizobacteria, plant–soil–microbiome responses, rhizosphere microbiome, root architecture, *Suillus luteus*

## Abstract

Beneficial fungal–bacterial interactions are important drivers of rhizosphere microecology and plant–soil functional coupling in conifer seedling systems, but their strain-combination-specific effects remain insufficiently understood. In this study, *Pinus sylvestris* var. *mongolica* seedlings were inoculated with three plant growth-promoting rhizobacteria (PGPR) strains, *Serratia plymuthica* A13, *Acinetobacter lwoffii* A07, and *Pseudomonas koreensis* A20, the ectomycorrhizal fungal strain *Suillus luteus* N94, and their corresponding co-inoculation combinations. Seedling growth, root architecture, plant nutrients, soil nutrients, soil enzyme activities, bacterial and fungal communities, differential taxa, network key taxa, and plant–soil functional indices were analyzed. Different inoculation treatments produced treatment- and trait-specific responses, with several N94–PGPR combinations showing advantages in particular growth, root, and soil functional traits, while some single-inoculation treatments also showed distinct positive effects. N94_A20 showed the greatest increases in seedling height, total dry weight, soil available phosphorus, and soil multifunctionality, whereas N94_A07 showed the strongest root architecture response and relative interaction index. Co-inoculation also reshaped rhizosphere bacterial and fungal communities and generated treatment-specific microbial enrichment patterns. *Massilia*, *Ramlibacter*, *Holtermanniella*, and *Naganishia* were positively associated with plant–soil functional indices. These results indicate that PGPR–N94 co-inoculation promotes conifer seedling growth through coordinated changes in root architecture, nutrient acquisition, soil biochemical function, and rhizosphere microbial community assembly.

## 1. Introduction

*Pinus sylvestris* var. *mongolica* is an important coniferous tree species for sandy land afforestation, windbreak establishment, sand fixation, and ecological restoration in northern China. It is characterized by strong tolerance to cold, drought, poor soil fertility, and wind stress, and has been widely used for vegetation restoration in semi-arid sandy lands and agro-pastoral ecotones [1]. Previous studies have shown that *P. sylvestris* var. *mongolica* plantations can increase vegetation coverage, improve soil physical structure and some nutrient conditions, and contribute to wind erosion reduction and sandy land restoration [2]. However, under arid, semi-arid, and nutrient-poor soil conditions, early seedling growth, root development, and nutrient acquisition of *P. sylvestris* var. *mongolica* may still be constrained by water and soil nutrient limitations [3]. Root architecture, nutrient uptake capacity, and the rhizosphere microenvironment at the seedling stage can affect seedling quality and the foundation for subsequent afforestation success [4]. Therefore, exploring biological regulation strategies that promote these seedling traits and rhizosphere functions is of practical importance for seedling cultivation and ecological restoration.

Rhizosphere microorganisms are important biological factors that influence plant growth and soil function [5]. Plant growth-promoting rhizobacteria, or PGPR, can promote plant growth through nutrient mobilization, phytohormone production, phosphate solubilization, stress alleviation, and regulation of the rhizosphere microbiome [6]. Recent studies further indicate that PGPR inoculation can positively affect plant phenotypes and soil biochemical properties and can also alter rhizosphere bacterial and fungal community structures, with different inoculated strains showing distinct effects on the rhizosphere microbiome [7]. Beneficial fungi, especially mycorrhizal fungi and other plant growth-promoting fungi, can expand the rhizosphere absorption interface through hyphal networks, promote plant acquisition of water and mineral nutrients, and participate in rhizosphere microbial interactions [8]. In the *P. sylvestris* var. *mongolica* system, ectomycorrhizal fungal inoculation and nitrogen supply have been shown to jointly affect seedling root morphology and nutrient uptake capacity [9]. These studies suggest that beneficial rhizosphere bacteria and fungi may serve as important microbial resources for regulating seedling growth and rhizosphere function in *P. sylvestris* var. *mongolica*.

Recent studies on the combined inoculation of arbuscular mycorrhizal fungi or other beneficial fungi with PGPR have shown that microbial co-inoculation can affect plant growth, nutrient acquisition, rhizosphere microecology, and soil biochemical responses in different plant systems [10]. Compared with single-strain inoculation, microbial co-inoculation may also better represent natural rhizosphere conditions, where multiple microbial taxa interact with plant roots and with each other. This advantage may arise from the division of functions and coordination between bacteria and fungi in rhizosphere resource use, nutrient transformation, root regulation, and microbial community assembly [11]. Among these processes, phosphorus transformation is an important component of co-inoculation effects. Phosphorus often exists in poorly soluble forms in soil, limiting plant uptake and utilization. Phosphate-solubilizing microorganisms can promote soil phosphorus transformation and plant phosphorus uptake through organic acid secretion, phosphatase production, root exudate interactions, and microbial community regulation [12]. Therefore, combining PGPR with beneficial fungi may provide an effective approach for improving root development, nutrient acquisition, and rhizosphere soil function in coniferous seedlings.

At present, studies on microbial co-inoculation have mainly focused on crops, horticultural plants, and herbaceous plant systems, whereas studies on the combined inoculation of PGPR and beneficial fungi in coniferous seedlings, especially *P. sylvestris* var. *mongolica*, remain relatively limited. Existing studies on *P. sylvestris* var. *mongolica* have mainly examined the effects of ectomycorrhizal fungi on root morphology, nutrient uptake, soil properties, and the rhizosphere microenvironment [13]. However, it remains unclear whether different PGPR strains combined with the same beneficial fungal strain produce consistent growth-promoting effects. Moreover, previous studies have often focused on single aspects such as growth or soil nutrients, while integrated responses involving plant growth, root architecture, plant nutrients, soil function, and rhizosphere bacterial and fungal communities have received less attention. Whether different co-inoculation combinations promote seedling growth through the same pathway or show strain-combination specificity also requires further clarification.

Based on these knowledge gaps, this study used *P. sylvestris* var. *mongolica* seedlings as the experimental material and established PGPR-alone inoculation, fungal strain *Suillus luteus* N94-alone inoculation, and co-inoculation treatments combining N94 with different PGPR strains. We systematically compared the effects of different treatments on seedling growth, root architecture, plant nutrient accumulation, soil nutrient availability, soil enzyme activities, and rhizosphere bacterial and fungal communities. This study aimed to answer the following questions: first, how PGPR-alone inoculation, N94-alone inoculation, and N94–PGPR co-inoculation differ in their effects on seedling growth and root development; second, whether different co-inoculation combinations differ in root architecture, nutrient accumulation, and soil function; and third, whether co-inoculation participates in plant–soil functional responses by reshaping rhizosphere bacterial and fungal communities and selecting key microbial taxa. Through these analyses, this study aimed to clarify the integrated plant–soil–microbiome response mechanism by which PGPR–N94 co-inoculation promotes conifer seedling growth, using *P. sylvestris* var. *mongolica* as a representative seedling system, and to provide a basis for screening microbial co-inoculation for seedling cultivation.

## 2. Materials and Methods

### 2.1. Plant Materials and Microbial Strains

The experimental plant was *Pinus sylvestris* var. *mongolica* seedlings. Seeds were purchased from the Zhanggutai Nursery Base, Zhangwu County, Liaoning Province, China. After germination, the seeds were sown in plastic pots. The growth substrate was prepared by mixing peat soil, vermiculite, and river sand at a volume ratio of 2:1:1. The substrate was sterilized at 121 °C for 2 h, placed into 15 cm × 15 cm pots, and kept at room temperature for 7 d before sowing. The sterilized substrate was prepared as a single homogeneous batch and uniformly distributed among pots to minimize differences in initial physicochemical background among treatments. No separate physicochemical analysis was conducted immediately after sterilization; therefore, the soil properties measured in this study represent the final rhizosphere status after inoculation and seedling growth rather than the initial post-sterilization baseline. Thirty seeds were sown in each pot and maintained in the greenhouse of the Experimental Forest Farm of Northeast Forestry University. After emergence, seedlings were thinned to 10 uniform individuals per pot and managed under routine greenhouse conditions. In the subsequent statistical analysis, each pot was regarded as one experimental unit, and the mean value of seedlings within each pot was used as one biological replicate.

The fungal strain used in this study was *Suillus luteus* N94, with the GenBank accession number MT652994. It was isolated from a *P. sylvestris* var. *mongolica* plantation at the Zhanggutai Experimental Forest Farm, Zhangwu County, Liaoning Province, China, and was identified based on rDNA sequence analysis and phylogenetic comparison. The PGPR strains used in this study are *Pseudomonas koreensis* A20, *Serratia plymuthica* A13, and *Acinetobacter lwoffii* A07. These bacterial isolates were previously screened by our laboratory from the rhizosphere soil of *P. sylvestris* var. *Mongolica* plantations at the Zhanggutai Experimental Forest Farm. This site is located in the sandy plantation region of northeastern China and is characterized by seasonal drought and marked soil moisture fluctuation [14]. The bacterial strains were purified and identified based on 16S rRNA gene sequencing and phylogenetic analysis. The GenBank accession numbers of A20, A13, and A07 were MT280201, MT280202, and MT280206, respectively. These strains were selected as representative PGPR inoculants because they showed plant growth-promoting potential in preliminary laboratory screening and were suitable for comparison with the ectomycorrhizal fungal strain N94 in the present co-inoculation experiment. All strains were preserved in the Forest Microbiology Laboratory of Northeast Forestry University.

The N94 strain stored at 4 °C was transferred onto PDA plates and cultured for 20 d. Mycelial plugs with a diameter of 10 mm were then cut using a sterile punch and inoculated into 500 mL Erlenmeyer flasks containing 250 mL of PD liquid medium. Three mycelial plugs were added to each flask. The cultures were incubated at 25 °C and 150 r min^−1^ for 30 d. Before inoculation, the mycelia were homogenized using a sterile blender to prepare the N94 inoculum.

Strains A13, A07, and A20 were separately streaked onto NB agar plates and incubated in the dark at 30 °C for 48 h. Single colonies were transferred into NB liquid medium and cultured at 28 °C and 180 r min^−1^ for 24 h. The cultures were centrifuged at 8000 r min^−1^ for 10 min at 4 °C. The supernatant was discarded, and the bacterial cells were washed three times with sterile water. Before inoculation, the bacterial suspension was adjusted to OD_600_ = 0.6, corresponding to approximately 1 × 10^8^ CFU mL^−1^. For co-inoculation treatments, the N94 inoculum and the corresponding PGPR suspension were mixed at equal volumes immediately before application.

### 2.2. Experimental Design and Inoculation Treatments

The experiment was arranged in a completely randomized design. Eight treatments were established: CK, A13, A07, A20, N94, N94_A13, N94_A07, and N94_A20. CK was the uninoculated control. A13, A07, and A20 were PGPR-alone treatments. N94 was the fungal-alone treatment. N94_A13, N94_A07, and N94_A20 were fungal–bacterial co-inoculation treatments consisting of N94 and the corresponding PGPR strain. The inoculation type, biological component, inoculum composition, and comparative role of each treatment are summarized in Table 1.

Inoculation was performed 14 d after seedling emergence. A hole-assisted root drenching method was used [15]. Five holes were made in the rhizosphere zone of each pot, with a diameter of 10 mm and a depth of 5 cm. Each pot received a total inoculation volume of 50 mL. For PGPR-alone treatments, 50 mL of the corresponding bacterial suspension was applied. For the N94-alone treatment, 50 mL of N94 fungal inoculum was applied. For co-inoculation treatments, 25 mL of N94 fungal inoculum and 25 mL of the corresponding PGPR suspension were mixed immediately before application. The CK treatment received 50 mL of sterile carrier solution without microbial inoculum. Thus, all treatments received the same total liquid volume, and the co-inoculation treatments were compared with single-inoculation treatments under an equal total inoculation volume.

Each treatment included six biological replicates, with one pot as one replicate. After inoculation, all pots were maintained under uniform greenhouse conditions at the Experimental Forest Farm of Northeast Forestry University. The positions of pots were periodically randomized to reduce the effects of spatial variation in light, temperature, and moisture. Plant and rhizosphere soil samples were collected 90 d after inoculation for subsequent analyses of seedling growth, root architecture, plant nutrients, soil nutrients, soil enzyme activities, and rhizosphere microbial communities.

### 2.3. Measurement of Seedling Growth, Root Architecture, and Relative Interaction Index

Plant samples were collected 90 d after inoculation. During sampling, the plastic pots were cut open, and the soil around the roots was carefully removed with a brush to minimize root damage. The roots were gently washed with sterile water and blotted dry with absorbent paper before growth and root trait measurements.

Seedling height was measured from the substrate surface to the top bud. Ground diameter was measured approximately 1 cm above the substrate surface using a vernier caliper. After harvest, the plants were divided into aboveground and belowground parts, or further separated into roots, stems, and leaves. The samples were dried at 75 °C to constant weight, and dry mass was recorded. When roots, stems, and leaves were separated, the dry mass of each organ was measured, and total dry weight was calculated. The mean value of seedlings within each pot was used as the replicate value.

Cleaned roots were spread in a transparent tray to avoid overlapping. Root images were obtained using an Epson V700 root scanner (Seiko Epson Corporation, Suwa, Japan). Root length, root surface area, average diameter, and root volume were quantified from the scanned images using WinRHIZO software (version 2013, Regent Instruments Inc., Quebec, QC, Canada), following previously described root image analysis procedures. These root traits were Z-score standardized and averaged to obtain a composite root architecture index.

The co-inoculation interaction effect was evaluated using the relative interaction index, RII [16]. In this study, RII was calculated based on the composite root architecture index to compare the root-enhancing effect of each N94_PGPR combination relative to the corresponding PGPR-alone treatment. The formula is

$$\mathrm{RII}=\frac{{RAI}_{N94\_\mathrm{PGPR}}-{\;\mathrm{RAI}}_{\mathrm{PGPR}}}{{RAI}_{N94\_\mathrm{PGPR}}+{\mathrm{RAI}}_{\mathrm{PGPR}}}$$

where${\;\mathrm{RAI}}_{N94\_\mathrm{PGPR}}$ represents the composite root architecture index of the N94_PGPR co-inoculation treatment, and ${\mathrm{RAI}}_{\mathrm{PGPR}}$ represents the composite root architecture index of the corresponding PGPR-alone treatment. N94_A13, N94_A07, and N94_A20 were compared with A13, A07, and A20, respectively. The N94-alone treatment was not included in the RII calculation because this index was designed to quantify the relative effect of adding N94 to each PGPR-alone treatment, rather than to evaluate the absolute effect of N94 alone. RII > 0 indicates a positive enhancement effect of co-inoculation relative to the corresponding PGPR-alone treatment, whereas RII < 0 indicates a weaker effect than the corresponding PGPR-alone treatment.

### 2.4. Measurement of Plant Nutrients, Soil Nutrients, and Soil Enzyme Activities

Dried plant samples were separated into roots, stems, and leaves and ground into powder for nutrient analysis. Total nitrogen (TN) was determined using the Kjeldahl method with a K9840 automatic Kjeldahl nitrogen analyzer (Hanon Instruments Co., Ltd., Jinan, China). Total phosphorus (TP) was determined using the molybdenum antimony colorimetric method. Total potassium (TK) was determined using the flame photometric method with an FP6430 flame photometer (INESA Analytical Instrument Co., Ltd., Shanghai, China). Organic matter (OM) was determined using the potassium dichromate external heating method. These nutrient measurements followed routine agrochemical analysis methods [17]. All plant nutrient indicators were Z-score standardized and averaged to obtain a composite plant nutrient index.

For rhizosphere soil sampling, roots were gently shaken, and the soil adhering to the root surface was collected into pre-sterilized sampling bags. Sampling tools were sterilized before use and disinfected with 75% ethanol between samples to reduce cross-contamination. Soil samples were passed through a 2 mm sieve and transported to the laboratory in an insulated box containing ice packs. Soil samples for nutrient analysis were air-dried and stored at room temperature. Samples for soil enzyme analysis were stored at 4 °C, and samples for microbial diversity analysis were stored at −80 °C.

Soil OM, TN, TP, and TK were determined using the same methods as those used for plant samples. Available nitrogen (AN) was determined using the alkaline hydrolysis diffusion method. Available phosphorus (AP) was determined using the double-acid extraction molybdenum antimony colorimetric method. Available potassium (AK) was determined using ammonium acetate extraction followed by flame photometry. Soil nutrient indicators were measured according to routine soil agrochemical analysis methods [17].

Soil acid phosphatase (S_Acp), catalase (S_Cat), sucrase (S_Sc), and urease (S_Ue) activities were measured using soil enzyme assay kits from Nanjing Jiancheng Bioengineering Institute (Nanjing, China), following the manufacturer’s instructions and standard soil enzyme assay procedures [17]. OM, AN, AP, AK, S_Acp, S_Cat, S_Sc, and S_Ue were Z-score standardized and averaged to obtain a soil multifunctionality index.

### 2.5. Rhizosphere Soil DNA Extraction, Amplicon Sequencing, and Sequence Processing

Total DNA was extracted from rhizosphere soil using the E.Z.N.A.^®^ Soil DNA Kit (Omega Bio-tek, Norcross, GA, USA). DNA concentration and purity were determined using a NanoDrop 2000 spectrophotometer (Thermo Fisher Scientific, Waltham, MA, USA), and DNA integrity was checked by 1% agarose gel electrophoresis. The V3–V4 region of the bacterial 16S rRNA gene was amplified using primers 341F and 806R. The primer sequences were 341F, 5′-ACTCCTACGGGAGGCAGCAG-3′, and 806R, 5′-GGACTACHVGGGTWTCTAAT-3′. The fungal rDNA ITS region was amplified using primers ITS1F and ITS2R. The primer sequences were ITS1F, 5′-ATATGCTTAAATTCAGCGGG-3′, and ITS2R, 5′-ATATGTAGGATGAAGAACGYAGAA-3′.

PCR products were checked and recovered by 2% agarose gel electrophoresis. The PCR products were purified using the AxyPrep DNA Gel Extraction Kit (Axygen Biosciences, Union City, CA, USA) and eluted with Tris-HCl. The purified products were checked again by 2% agarose gel electrophoresis. The purified amplicons were quantified using QuantiFluor™-ST (Promega Corporation, Madison, WI, USA), mixed in appropriate proportions according to sequencing requirements, and used to construct MiSeq libraries. Sequencing was performed on the Illumina MiSeq platform (Illumina, Inc., San Diego, CA, USA).

Raw sequences were merged using FLASH (version 1.2.11) and quality-filtered using Trimmomatic (version 0.39) [18]. The quality-filtered sequences were analyzed using UPARSE (version 7.1) and clustered into operational taxonomic units (OTUs) at 97% sequence similarity [19]. Chimeric sequences were removed using UCHIME (version 4.2) [20]. Taxonomic classification was performed using the RDP Classifier (version 2.2) with a confidence threshold of 0.7 [21]. Bacterial and fungal communities were used for subsequent analyses of alpha diversity, beta diversity, community composition, differential enrichment, and co-occurrence networks. Alpha diversity was evaluated using the Chao index. Beta diversity was calculated based on Bray–Curtis distances and visualized using non-metric multidimensional scaling (NMDS). Group differences were tested using ANOSIM or PERMANOVA with 999 permutations.

### 2.6. Differential Enrichment Analysis, Network Analysis, and Candidate Key Taxa Screening

Differentially enriched taxa were identified using LEfSe analysis. The screening criteria were Kruskal–Wallis test *p* < 0.05 and LDA score > 3. The analysis was performed separately at the bacterial and fungal genus levels.

Co-occurrence networks were constructed separately for bacterial and fungal communities. Spearman correlation analysis was used to calculate associations among taxa, and the Benjamini–Hochberg method was used for FDR correction [22]. Network topological parameters were calculated using igraph [23]. Zi-Pi analysis was used to classify peripheral nodes, connectors, and module hubs. Structural keystoneness analysis was used to identify potential keystone OTUs that contributed strongly to network structure.

Candidate key taxa were selected based on three lines of evidence: LEfSe-enriched genera, genera corresponding to the top 10 keystone OTUs, and genera corresponding to Zi-Pi connectors or module hubs. The final candidate taxa were used for Mantel tests and correlation analysis with plant–soil functional indices. Network analysis results were interpreted only as statistical associations and not as direct evidence of causality.

### 2.7. Plant–Soil Functional Indices and Microbial Functional Association Analysis

Five composite indices were constructed to integrate plant and soil functional responses. PGI represented the plant growth index and included seedling height, ground diameter, and total dry weight. RAI represented the root architecture index and included root length, root surface area, average diameter, and root volume. PNI represented the plant nutrient index and included TN, TP, and TK in roots, stems, and leaves. SNAI represented the soil nutrient availability index and included OM, AN, AP, and AK. SEAI represented the soil enzyme activity index and included S_Acp, S_Cat, S_Sc, and S_Ue. All indicators were first Z-score standardized and then averaged within each category.

Mantel tests were used to evaluate the relationships between candidate key taxa and soil properties. Soil variables included OM, AN, AP, AK, S_Acp, S_Cat, S_Sc, and S_Ue. The number of permutations was set to 999. Treatment-level Spearman correlation analysis was used to examine the relationships between candidate microbial taxa and the five composite indices, followed by FDR correction. Correlation results were used to reflect associations between candidate taxa and plant–soil functional status and were not interpreted as evidence of causality.

### 2.8. Statistical Analysis

All statistical analyses were performed using pots as the basic experimental units. Before analysis, data normality and homogeneity of variance were examined using the Shapiro–Wilk test and Levene’s test, respectively. Data that met the assumptions of parametric tests were analyzed using one-way analysis of variance, followed by Duncan’s multiple range test for post hoc comparisons. Data that did not meet parametric assumptions were analyzed using the Kruskal–Wallis test, followed by Dunn’s multiple comparison test. The significance level was set at *p* < 0.05. Correlation analysis was performed using Spearman’s method, and FDR correction was conducted using the Benjamini–Hochberg method [22].

Statistical analyses were performed using SPSS 25.0 and R software version 4.0.3 [24]. The vegan, ggplot2, pheatmap, igraph, Hmisc, and psych packages were mainly used for statistical analysis and visualization. Figures were prepared using OriginPro 9.0, Origin 2021. All results from differential enrichment analysis, network nodes, and correlation analysis were interpreted as statistical associations. Further validation through isolation, re-inoculation experiments, and colonization dynamics analysis is still needed.

## 3. Results and Analysis

### 3.1. Effects of Microbial Inoculation on Seedling Growth and Root Morphology

**Figure 1:**
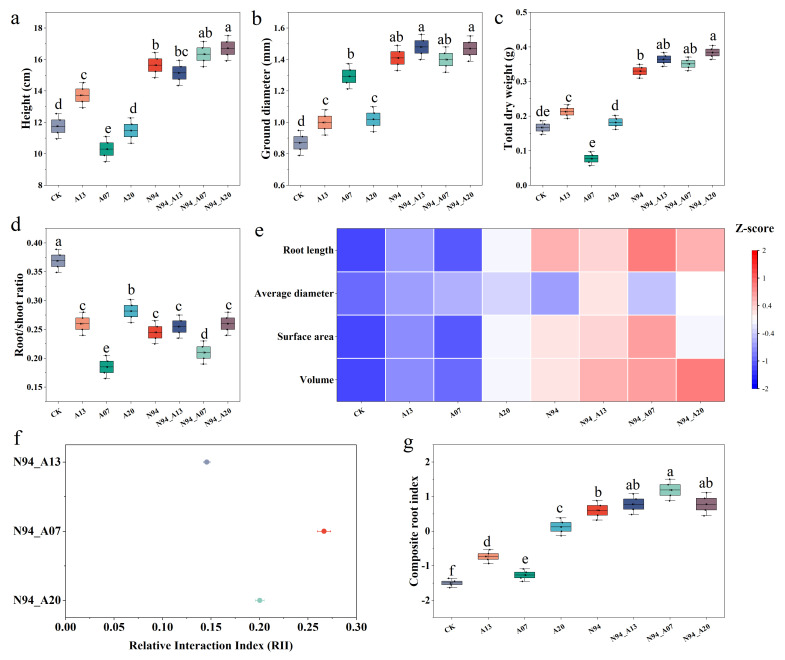
Effects of PGPR, fungal strain N94, and their co-inoculation on seedling growth and root development of *Pinus sylvestris* var. *mongolica*. (**a**–**c**) Seedling height, ground diameter, and total dry weight. (**d**) Root/shoot ratio. (**e**) Z-score heatmap of root architectural traits. (**f**) Relative Interaction Index (RII) for co-inoculation treatments. (**g**) Composite root index. CK, uninoculated control; A13, A07, and A20, PGPR-alone treatments; N94, fungal strain N94 alone; N94_A13, N94_A07, and N94_A20, co-inoculation treatments. Different lowercase letters indicate significant differences among treatments at *p* < 0.05. Different colors represent different inoculation treatments.

Different inoculation treatments markedly significantly affected the growth and root development of *Pinus sylvestris* var. *mongolica* seedlings (Figure 1). Compared with CK, most inoculation treatments increased seedling height, ground diameter, and total dry weight to different extents, but the dominant responses differed among treatments. For seedling height, N94_A20 showed the highest value, followed by N94_A07, whereas N94 also showed a significant increase compared with CK. For ground diameter, N94_A13 and N94_A20 showed relatively high values, and N94 and N94_A07 were also maintained at a high level. For total dry weight, N94_A20 showed the strongest response, while N94_A13 and N94_A07 were also higher than most single-PGPR treatments. These results indicate that seedling growth responses were treatment-specific, and the positive effects were not restricted to co-inoculation treatments.

Root development also showed distinct treatment-dependent patterns. The root/shoot ratio was highest in CK, whereas inoculated treatments generally showed lower ratios, suggesting that inoculation changed biomass allocation between aboveground and belowground parts. The root trait heatmap showed that N94-containing treatments generally improved root architectural traits, but the dominant root traits differed among combinations. N94_A07 showed higher values for root length and root surface area, while N94_A20 was associated with higher root volume. The composite root index further showed that N94_A07 had the highest value, followed by N94_A13 and N94_A20, whereas N94 alone also showed a higher root index than PGPR-alone treatments. The RII values of N94_A13, N94_A07, and N94_A20 were all positive, indicating that adding N94 to the corresponding PGPR treatment produced positive relative responses in the composite root architecture index. Among the three co-inoculation treatments, N94_A07 showed the highest RII. Overall, inoculation effects on seedling growth and root architecture were treatment- and trait-specific rather than uniformly stronger in all co-inoculation treatments.

### 3.2. Effects of Microbial Inoculation on Plant Nutrient Accumulation

**Figure 2:**
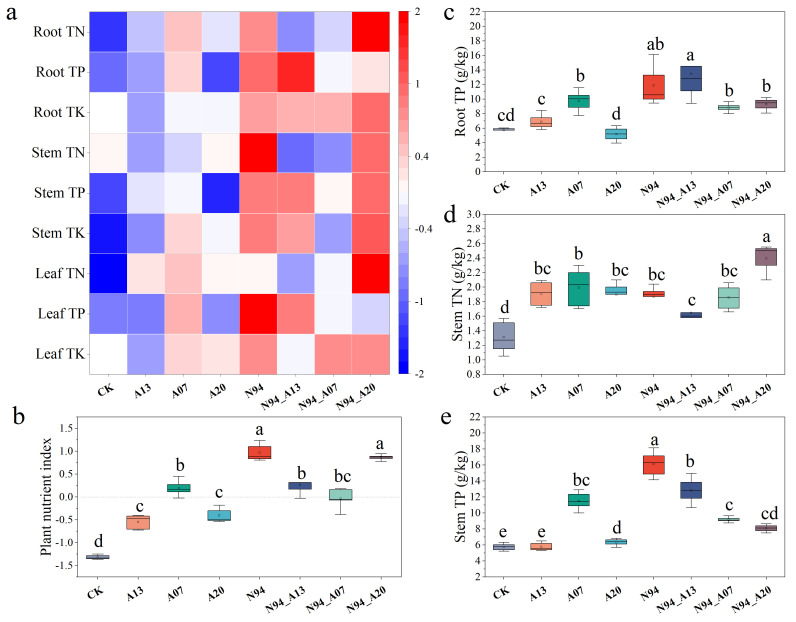
Effects of PGPR, fungal strain N94, and their co-inoculation on nutrient accumulation in *Pinus sylvestris* var. *mongolica* seedlings. (**a**) Z-score heatmap showing total nitrogen (TN), total phosphorus (TP), and total potassium (TK) contents in roots, stems, and leaves under different inoculation treatments. Red and blue indicate higher and lower standardized values, respectively. (**b**) Plant nutrient index integrating TN, TP, and TK contents across roots, stems, and leaves. (**c**–**e**) Representative nutrient indicators showing root TP, stem TN, and stem TP contents. CK, uninoculated control; A13, A07, and A20, PGPR-alone treatments; N94, fungal strain N94 alone; N94_A13, N94_A07, and N94_A20, co-inoculation treatments. Boxplots show the distribution of biological replicates, with dots representing individual replicate values. Different lowercase letters indicate significant differences among treatments at *p* < 0.05. Different colors represent different inoculation treatments.

Different inoculation treatments changed nutrient accumulation patterns in roots, stems, and leaves of *P. sylvestris* var. *mongolica* seedlings (Figure 2). The Z-score heatmap showed clear differences in TN, TP, and TK among treatments and plant organs. Compared with CK, most inoculation treatments improved plant nutrient status to different extents; however, the dominant responses differed among single-inoculation and co-inoculation treatments as well as among plant organs.

For root nutrients, root TP was higher in N94 and N94_A13, while N94_A20 also showed a relatively high overall nutrient status in the heatmap. For stem nutrients, N94 showed high levels of stem TN, stem TP, and stem TK, whereas N94_A20 had the highest stem TN. N94_A13 also maintained a relatively high stem TP level, indicating different effects of N94-containing treatments on stem nutrient accumulation. For leaf nutrients, N94_A20 showed stronger increases in leaf TN and leaf TK, while N94 and N94_A13 showed higher leaf TP.

The plant nutrient index further integrated the nutrient responses across different organs and elements. N94 and several co-inoculated treatments showed relatively high overall plant nutrient status, but this response was not restricted to co-inoculation. Notably, the A07 single-inoculation treatment also showed a marked plant nutrient response and was comparable to, or higher than, some co-inoculation treatments, including N94_A07. This indicates that single PGPR inoculation may contribute strongly to nutrient accumulation in a treatment-specific manner. Among the co-inoculated groups, N94_A20 showed a higher plant nutrient index and stronger responses in leaf TN and TK, whereas N94_A13 contributed more strongly to root and stem phosphorus accumulation. These results indicate that inoculation effects on plant nutrient acquisition and allocation were compartmentalized and treatment-specific rather than representing a uniformly stronger response of co-inoculation treatments.

### 3.3. Effects of Microbial Inoculation on Rhizosphere Soil Nutrients and Enzyme Activities

**Figure 3:**
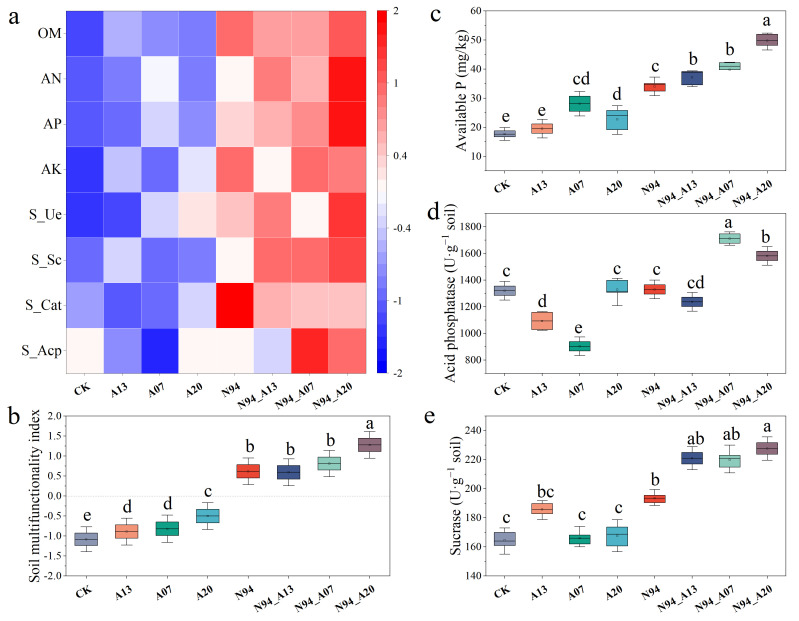
Effects of PGPR, fungal strain N94, and their co-inoculation on soil nutrient availability, enzyme activities, and soil multifunctionality in the rhizosphere of *Pinus sylvestris* var. *mongolica* seedlings. (**a**) Z-score heatmap showing soil organic matter (OM), available nitrogen (AN), available phosphorus (AP), available potassium (AK), urease activity (S_Ue), sucrase activity (S_Sc), catalase activity (S_Cat), and acid phosphatase activity (S_Acp) under different inoculation treatments. Red and blue indicate higher and lower standardized values, respectively. (**b**) Soil multifunctionality index integrating soil nutrient availability and enzyme activity indicators. (**c**–**e**) Representative soil functional indicators showing available P, acid phosphatase activity, and sucrase activity. CK, uninoculated control; A13, A07, and A20, PGPR-alone treatments; N94, fungal strain N94 alone; N94_A13, N94_A07, and N94_A20, co-inoculation treatments. Boxplots show the distribution of biological replicates, with dots representing individual replicate values. Different lowercase letters indicate significant differences among treatments at *p* < 0.05. Different colors represent different inoculation treatments.

Inoculation treatments changed rhizosphere soil nutrient availability, enzyme activities, and the soil multifunctionality index (Figure 3). The Z-score heatmap showed that OM, AN, AP, AK, S_Ue, S_Sc, S_Cat, and S_Acp responded differently among treatments, indicating that soil nutrient and enzyme responses were not synchronized across all indicators. Compared with CK, most inoculation treatments improved one or more soil functional indicators, but the dominant soil responses differed among PGPR-alone, N94-alone, and co-inoculation treatments.

For soil nutrient availability, N94_A20 showed a pronounced increase in AP and had a high soil multifunctionality index. N94 and N94_A07 also showed relatively high AP levels, whereas A07 displayed a positive response in several soil-related indicators. OM followed a similar treatment-dependent trend to other soil nutrient variables, indicating that changes in soil organic matter were also involved in the overall soil functional response.

Soil enzyme activities showed patterns that were not completely consistent with soil nutrient availability. Although N94_A20 showed the highest AP response, the highest S_Acp activity was observed in N94_A07. This indicates that higher phosphatase activity did not directly correspond to the highest available phosphorus level across treatments. In contrast, the responses of nitrogen-related and carbon-related indicators showed different coordination patterns, with S_Ue and S_Sc contributing to the overall soil biochemical response. S_Cat also varied among treatments. Overall, the soil multifunctionality index showed that N94_A20 had the most pronounced integrated soil response, but individual soil nutrients and enzyme activities differed among treatments.

### 3.4. Rhizosphere Microbial Diversity and Community Composition in Sterilized Substrate

**Figure 4:**
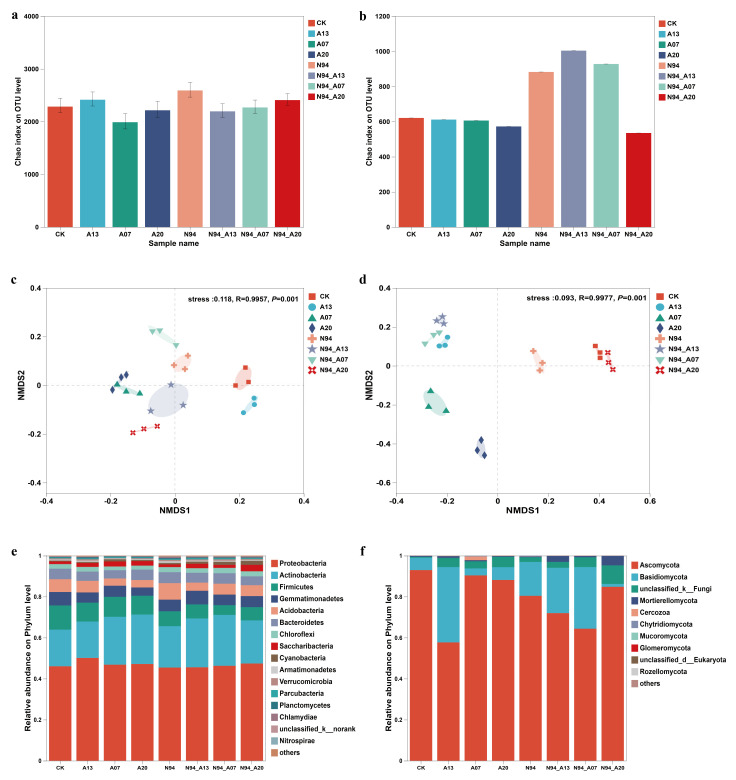
Effects of PGPR, fungal strain N94, and their co-inoculation on rhizosphere bacterial and fungal community diversity, structure, and composition in *Pinus sylvestris* var. *mongolica* seedlings. (**a**) Chao richness index of the bacterial community at the OTU level. (**b**) Chao richness index of the fungal community at the OTU level. (**c**) NMDS ordination of the bacterial community based on Bray–Curtis distance. The stress value and ANOSIM statistics are shown in the plot. (**d**) NMDS ordination of the fungal community based on Bray–Curtis distance. The stress value and ANOSIM statistics are shown in the plot. (**e**) Relative abundance of dominant bacterial phyla across different inoculation treatments. (**f**) Relative abundance of dominant fungal phyla across different inoculation treatments. CK, uninoculated control; A13, A07, and A20, PGPR-alone treatments; N94, fungal strain N94 alone; N94_A13, N94_A07, and N94_A20, co-inoculation treatments.

Inoculation treatments changed bacterial and fungal richness, community structure, and phylum-level composition in the rhizosphere of seedlings grown in sterilized substrate (Figure 4). For bacterial richness, the Chao index was highest in N94, followed by N94_A20 and A13. For fungal richness, N94_A13 and N94_A07 showed higher Chao values, whereas N94_A20 had the lowest fungal Chao value among all treatments. These results showed that bacterial and fungal richness responded differently to the inoculation treatments.

In the bacterial community, the Chao index varied among treatments, with N94 showing the highest richness, followed by N94_A20 and A13. However, bacterial richness did not show a simple co-inoculation-dominant pattern, because A13 also showed a relatively high bacterial Chao value. In contrast, fungal richness showed a different pattern from bacterial richness. N94_A13 and N94_A07 had higher fungal Chao values, whereas N94_A20 showed the lowest fungal richness among all treatments. This result indicates that bacterial and fungal recolonization responded differently to the inoculation treatments.

NMDS analysis showed treatment-associated separation in both bacterial and fungal communities. The bacterial community showed partial separation among treatments, whereas the fungal community showed a clearer separation pattern.

At the phylum level, bacterial communities were mainly composed of Proteobacteria, Actinobacteria, Firmicutes, Gemmatimonadetes, Acidobacteria, and Bacteroidetes. Different inoculation treatments changed the relative abundance of these dominant bacterial phyla, but bacterial communities showed relatively limited separation from CK compared with fungal communities. Fungal communities were mainly composed of Ascomycota and Basidiomycota, and their relative proportions varied greatly among treatments. N94_A13 showed a relatively higher contribution of Basidiomycota, whereas N94_A20 was dominated by Ascomycota and showed the lowest fungal richness. These results indicate that fungal recolonization was more sensitive to inoculation regime than bacterial recolonization under the sterilized-substrate conditions. Detailed statistics for bacterial and fungal diversity and community structure are provided in Supplementary Table S1.

### 3.5. Differentially Enriched Bacterial and Fungal Genera Under Different Inoculation Treatments

**Figure 5:**
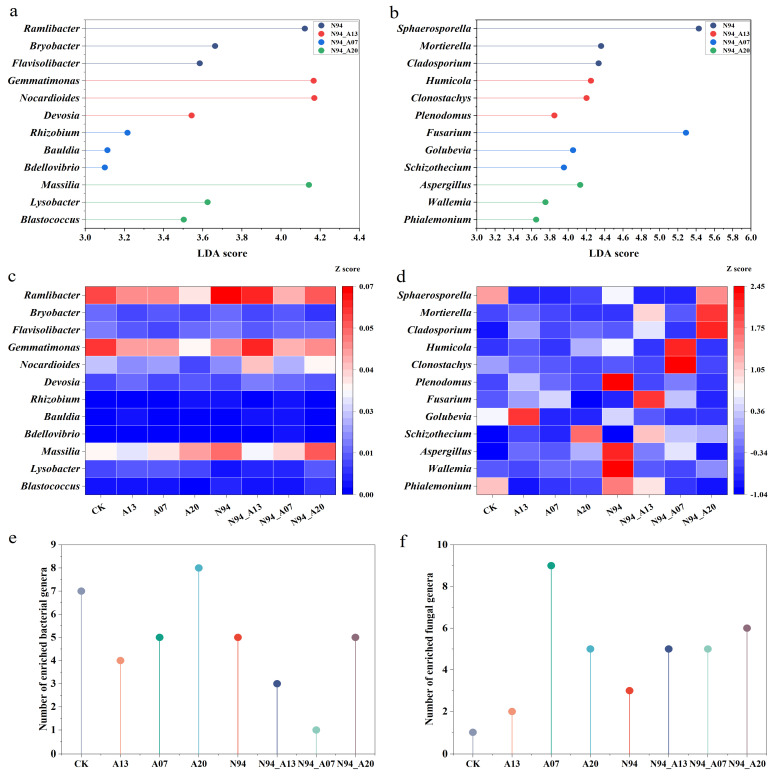
Differentially enriched bacterial and fungal genera under different inoculation treatments in the rhizosphere of *Pinus sylvestris* var. *mongolica* seedlings. (**a**) LEfSe-derived LDA scores of differentially enriched bacterial genera in the N94, N94_A13, N94_A07, and N94_A20 treatments. (**b**) LEfSe-derived LDA scores of differentially enriched fungal genera in the N94, N94_A13, N94_A07, and N94_A20 treatments. (**c**) Heatmap showing the relative abundance patterns of selected differentially enriched bacterial genera across all treatments. (**d**) Heatmap showing the relative abundance patterns of selected differentially enriched fungal genera across all treatments. (**e**) Number of enriched bacterial genera detected in each treatment. (**f**) Number of enriched fungal genera detected in each treatment. CK, uninoculated control; A13, A07, and A20, PGPR-alone treatments; N94, fungal strain N94 alone; N94_A13, N94_A07, and N94_A20, co-inoculation treatments. Differential taxa were identified using LEfSe analysis with an LDA score > 3. The results indicate that different N94–PGPR combinations recruited distinct bacterial and fungal indicator genera.

LEfSe analysis identified distinct bacterial and fungal genera enriched under different treatments (Figure 5). In the bacterial community, N94 and the three co-inoculated treatments were associated with distinct enriched genera. N94 was mainly characterized by *Ramlibacter*, *Bryobacter*, and *Flavisolibacter*. N94_A13 was mainly enriched in *Gemmatimonas*, *Nocardioides*, and *Devosia*. N94_A07 was mainly enriched in *Rhizobium*, *Bauldia*, and *Bdellovibrio*. N94_A20 was mainly enriched in *Massilia*, *Lysobacter,* and *Blastococcus*. In addition to N94-containing treatments, PGPR-alone treatments also showed treatment-specific bacterial enrichment. In particular, A20 had the highest number of enriched bacterial genera, including functionally relevant genera such as *Bacillus* and *Pseudomonas*.

Fungal differential genera also varied among treatments. N94 was mainly enriched in *Sphaerosporella*, *Mortierella*, and *Cladosporium*. N94_A13 was enriched in *Humicola*, *Clonostachys*, and *Plenodomus*. N94_A07 was characterized by the enrichment of *Fusarium*, *Golubevia*, and *Schizothecium*, while N94_A20 was enriched in *Aspergillus*, *Wallemia*, and *Phialemonium*. Among the PGPR-alone treatments, A07 showed the highest number of enriched fungal genera, including *Penicillium*, *Talaromyces*, and *Trichoderma*. Overall, the number and composition of enriched bacterial and fungal genera differed among inoculation treatments, and co-inoculated treatments did not consistently show the highest number of enriched genera. The enrichment of *Fusarium* in N94_A07 was noted as a potentially unfavorable fungal shift. The complete LEfSe results are provided in Supplementary Table S2.

### 3.6. Network Topology and Soil-Property Associations of Rhizosphere Microbial Communities

**Figure 6:**
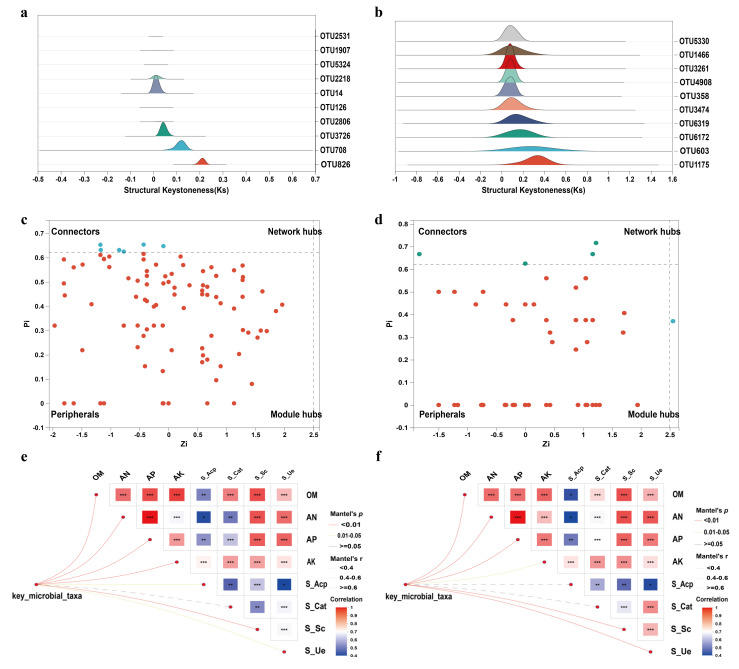
Potential keystone taxa, Zi-Pi topological roles, and soil functional associations of rhizosphere bacterial and fungal communities in *Pinus sylvestris* var. *mongolica* seedlings. (**a**) Structural keystoneness distribution of the top bacterial OTUs. Higher Ks values indicate greater potential contribution to bacterial network structure. (**b**) Structural keystoneness distribution of the top fungal OTUs. Higher Ks values indicate greater potential contribution to fungal network structure. (**c**) Zi-Pi plot showing the topological roles of bacterial network nodes. Nodes were classified as peripherals, connectors, module hubs, or network hubs according to within-module connectivity (Zi) and among-module connectivity (Pi). (**d**) Zi-Pi plot showing the topological roles of fungal network nodes. Nodes were classified using the same Zi-Pi criteria. (**e**) Mantel test linking candidate key bacterial taxa with soil nutrient and enzyme variables, together with pairwise correlations among soil properties. (**f**) Mantel test linking candidate key fungal taxa with soil nutrient and enzyme variables, together with pairwise correlations among soil properties. Soil variables included organic matter (OM), available nitrogen (AN), available phosphorus (AP), available potassium (AK), acid phosphatase activity (S_Acp), catalase activity (S_Cat), sucrase activity (S_Sc), and urease activity (S_Ue). Line color indicates Mantel’s *p* value, line width indicates Mantel’s r, and solid or dashed lines indicate positive or negative relationships, respectively. Heatmap colors indicate Spearman correlations among soil variables. *p* < 0.05, *p* < 0.01, and *p* < 0.001. Asterisks indicate significance levels: * *p* < 0.05, ** *p* < 0.01, and *** *p* < 0.001. Line color indicates Mantel’s *p* value, and heatmap colors indicate Spearman correlation coefficients among soil variables. Solid and dashed lines indicate positive and negative relationships, respectively.

Structural keystoneness and Zi-Pi analyses were used to identify candidate network-relevant taxa in bacterial and fungal communities (Figure 6). Most bacterial and fungal nodes were classified as peripherals, whereas only a small number of nodes were identified as connectors or module hubs. The top-ranked bacterial nodes showed relatively low structural keystoneness values. One bacterial node was annotated only as Cyanobacteria at the phylum level, and this annotation was clarified in Supplementary Table S3. In the fungal network, several top-ranked nodes were assigned to *Fusarium*.

Mantel tests showed associations between candidate network-relevant taxa and soil nutrient or enzyme variables, including OM, AN, AP, AK, S_Acp, S_Cat, S_Sc, and S_Ue. Bacterial and fungal taxa showed different association patterns with soil properties. Candidate bacterial taxa were mainly associated with nutrient-related indicators, whereas candidate fungal taxa showed stronger links with several enzyme-related variables. These results indicated that the network-relevant bacterial and fungal taxa were linked to different soil functional indicators. Because the substrate was sterilized before sowing, these relationships were interpreted as statistical associations within recolonized rhizosphere communities. Detailed information on the top candidate network-relevant OTUs and Zi-Pi topological nodes is provided in Supplementary Table S3.

### 3.7. Correlations Between Candidate Microbial Taxa and Plant–Soil Functional Indices

Table 2 presents the treatment-level Spearman correlations between candidate microbial taxa and five plant–soil functional indices, including the plant growth index, root architecture index, plant nutrient index, soil nutrient availability index, and soil enzyme activity index.

In the bacterial community, *Ramlibacter* was positively correlated with the plant growth index, whereas *Massilia* was positively correlated with the plant nutrient index and soil enzyme activity index. In contrast, *Tumebacillus* and *Oxalophagus* showed negative correlations with the root architecture index, soil nutrient availability index, and soil enzyme activity index. *Variovorax* and *Methylorosula* also showed negative correlations with several plant–soil functional indices, especially the root architecture index and soil nutrient availability index.

Fungal candidate taxa showed a different correlation pattern. *Holtermanniella*, identified as a Zi-Pi connector, was positively correlated with all five functional indices, with the strongest correlation observed for RAI. *Naganishia* was positively correlated with PNI. In contrast, *Talaromyces* was negatively correlated with PGI. Other fungal taxa, including *Fusarium*, *Tuber*, *Tomentella*, *Fusicolla*, *Sphaerosporella*, *Mortierella*, and *Clonostachys*, showed no significant correlations with the five functional indices. The complete Spearman correlation results, including *p* values and FDR-adjusted *p* values, are provided in Supplementary Table S4.

## 4. Discussion

### 4.1. Growth and Root Responses to PGPR-Alone, N94-Alone, and Fungal–Bacterial Co-Inoculation Treatments

The present study showed that PGPR alone, N94 alone, and N94–PGPR co-inoculation treatments all affected the growth of *Pinus sylvestris* var. *mongolica* seedlings to different extents. Rather than showing a uniform advantage of one inoculation type across all traits, the results revealed treatment- and trait-specific responses. N94_A20 showed the most pronounced increases in seedling height and total dry weight, whereas N94_A13 performed better in ground diameter. Meanwhile, N94-alone and several PGPR-alone treatments also showed positive effects on specific traits, indicating that single-inoculation responses should be considered when interpreting the overall inoculation effects. Recent studies have shown that co-inoculation of beneficial fungi and plant growth-promoting bacteria can simultaneously affect plant growth, rhizosphere microbial structure, and soil function, with the direction and magnitude of these effects depending on plant species, microbial partners, and environmental conditions [10].

The trait-specific effects of co-inoculation may be related to the complementary rhizosphere functions of PGPR and N94. PGPR can promote plant growth through nutrient mobilization, phytohormone production, increased phosphorus availability, and regulation of rhizosphere microbial communities [25]. Beneficial fungi can expand the rhizosphere absorption interface through hyphal networks and improve plant acquisition of mineral nutrients and water [8]. When N94 and PGPR were co-inoculated, the two microbial groups may have enhanced resource acquisition and functional coordination through rhizosphere and hyphosphere interactions [26].

However, the three co-inoculated treatments did not show identical advantages. N94_A07 showed stronger responses in RII and root architecture, whereas N94_A20 was more prominent in biomass accumulation. This indicates that co-inoculation was not a simple additive effect, but was influenced by the specific strain combination. Previous studies have shown that the effects of microbial inoculation are jointly regulated by strain compatibility, host plants, soil conditions, and the resident rhizosphere microbiome [27,28]. In this study, single-inoculation treatments also produced distinct positive responses in specific traits, whereas co-inoculation treatments showed different advantages depending on the bacterial partner combined with N94. This pattern suggests that microbial inoculation effects were determined by both the inoculated strain and the target plant trait, rather than by inoculation type alone. This difference may be related to the species characteristics, root development pattern, and rhizosphere microbiome assembly process of *P. sylvestris* var. *mongolica* seedlings [29,30].

### 4.2. Root Architecture as a Key Component of Seedling Growth Responses

Root architecture was one of the traits that responded strongly to co-inoculation in this study. Compared with CK and most single-inoculation treatments, co-inoculation generally increased root length, root surface area, root volume, and the composite root index. N94_A07 showed the strongest responses in root length, root surface area, RII, and the composite root index. Root architecture determines the ability of plants to explore soil space and directly affects water and mineral nutrient uptake efficiency [31]. Therefore, the pronounced improvement of root architecture under N94_A07 may partly explain its higher relative interaction index.

Increases in root length and root surface area can enlarge the contact area between roots and soil and improve nutrient uptake opportunities [32]. Phosphorus has low mobility in soil, and plant phosphorus acquisition strongly depends on root exploration capacity and rhizosphere phosphorus transformation. Phosphate-solubilizing bacteria can improve plant phosphorus uptake through phosphate solubilization, organic phosphorus mineralization, phosphatase activation, and regulation of root structure [33]. In this study, N94_A07 mainly showed advantages in root architecture, whereas N94_A20 was more prominent in soil AP and soil multifunctionality. This suggests that different co-inoculation combinations may preferentially affect root expansion or soil nutrient transformation.

Previous studies on *P. sylvestris* var. *mongolica* have shown that ectomycorrhizal fungal inoculation and nitrogen supply can jointly affect root morphology and nutrient uptake capacity [34]. This is consistent with our finding that treatments involving N94 altered root architecture and plant nutrient status. However, the strongest root response was observed in N94_A07 rather than N94 alone, suggesting that the effect of N94 on root architecture may be more fully expressed when combined with a specific PGPR strain. In addition, N94_A07 showed the strongest root architecture and RII, whereas N94_A20 showed greater seedling height and total dry weight. This indicates that root architectural improvement was an important component of seedling growth responses, but it was not the only determining factor.

### 4.3. Plant Nutrient Accumulation and Soil Functional Responses Under Microbial Inoculation

Microbial inoculation changed nutrient accumulation in roots, stems, and leaves of *P. sylvestris* var. *mongolica* seedlings. N94 and several N94-containing treatments showed high nutrient accumulation in different plant organs, indicating that fungal inoculation and fungal–bacterial co-inoculation affected nutrient uptake and allocation. However, the response was not restricted to N94-containing treatments. The A07 single-inoculation treatment also showed a marked plant nutrient response, which suggests that this PGPR strain may have contributed to nutrient acquisition independently of N94. Beneficial rhizosphere bacteria and fungi are involved in plant acquisition of nitrogen, phosphorus, potassium, and other mineral nutrients. Bacteria mainly contribute through nutrient solubilization, mineralization, and rhizosphere environmental regulation, whereas fungi influence nutrient transport through hyphal absorption interfaces and symbiotic interactions [12]. Therefore, the nutrient responses observed in this study likely reflected both single-strain effects and PGPR–N94 combination effects.

N94 and all three co-inoculated treatments increased soil AP, with N94_A20 showing the strongest response. Phosphorus is easily fixed in soil, whereas phosphate-solubilizing microorganisms can improve soil phosphorus availability through organic acid secretion, phosphatase production, and rhizosphere interactions [35]. Bacteria and fungi have complementary roles in plant phosphorus acquisition, and mixed microbial communities can enhance plant phosphorus uptake [36]. Therefore, the increase in AP and TP accumulation in some plant organs suggests that microbial inoculation may strengthen the linkage between rhizosphere phosphorus transformation and plant phosphorus uptake.

Soil enzyme activities showed that soil biochemical responses were not fully synchronized with nutrient availability. Although N94_A20 had the highest soil AP and soil multifunctionality index, the highest acid phosphatase activity occurred in N94_A07. This indicates that higher phosphatase activity did not necessarily correspond to the highest available phosphorus level across treatments. Soil enzymes participate in organic matter decomposition and nutrient cycling. Phosphatases are associated with organic phosphorus mineralization, urease is involved in nitrogen transformation, and sucrase is closely related to carbon cycling [37,38]. Thus, plant nutrient accumulation, soil AP, and enzyme activities should be interpreted as related but not identical indicators of plant–soil functional responses.

### 4.4. Rhizosphere Microbial Recolonization and Community Composition in Sterilized Substrate

Different inoculation treatments changed the richness, community structure, and phylum-level composition of recolonized rhizosphere bacterial and fungal communities in sterilized substrate. The bacterial Chao index was higher in N94 and N94_A20, whereas the fungal Chao index was higher in N94_A13 and N94_A07. NMDS analysis showed treatment-related separation in both bacterial and fungal communities, with a more pronounced separation in the fungal community. Previous studies have shown that PGPR inoculation can alter rhizosphere microbiome structure, and that different inoculated strains may have different effects on rhizosphere communities [39]. This is consistent with the distinct community distribution patterns observed among different inoculation treatments in this study.

The responses of bacterial and fungal communities were not fully synchronized. N94_A20 showed relatively high bacterial richness, but did not show the highest fungal richness. N94_A13 and N94_A07 showed higher fungal richness, but their advantages in growth and soil function differed. Similar studies have shown that microbial inoculants can simultaneously alter bacterial and fungal community structures in the rhizosphere and cause treatment-dependent changes in dominant taxa [40]. Therefore, the different responses of bacteria and fungi to co-inoculation may be related to differences in rhizosphere niches, resource-use patterns, and microbial interactions.

Changes in community richness did not directly represent growth-promoting strength. N94_A20 did not have the highest fungal Chao index, but it showed stronger responses in seedling height, total dry weight, soil AP, and soil multifunctionality. Recent studies have suggested that plant production is often supported by functionally adapted core microbial assemblages rather than simply by an increase in microbial richness [41]. Rhizosphere effects can also strongly alter microbial community composition and beta diversity, indicating that community structure itself is important for understanding rhizosphere function [42]. Therefore, the microbial results in this study should be interpreted by considering both community composition and candidate taxa, rather than by Chao richness alone.

### 4.5. Differentially Enriched Taxa and Candidate Network-Relevant Taxa Associated with Plant–Soil Functional Responses

LEfSe analysis showed that different inoculation treatments corresponded to differentially enriched bacterial and fungal genera. The co-inoculated treatments were associated with distinct enriched taxa, such as *Massilia* and *Lysobacter* in N94_A20 and *Rhizobium* and *Bdellovibrio* in N94_A07. However, enriched taxa were not restricted to co-inoculation treatments. The A20 single-inoculation treatment showed enrichment of bacterial genera such as *Bacillus* and *Pseudomonas*, whereas the A07 single-inoculation treatment showed enrichment of fungal genera such as *Penicillium*, *Talaromyces*, and *Trichoderma*. In addition, *Fusarium* was enriched in N94_A07, suggesting that not all fungal shifts should be interpreted as favorable. Previous studies have shown that the rhizosphere can selectively enrich microbial taxa adapted to plant traits and resource environments [43].

Among candidate bacterial taxa, *Massilia* and *Ramlibacter* were positively correlated with some plant–soil functional indices. In particular, *Massilia* was positively correlated with the plant nutrient index and soil enzyme activity index. Previous studies have reported that *Massilia* can be enriched in the rhizosphere and is associated with plant growth, nitrogen accumulation, or rhizosphere nutrient use [44], which is consistent with the present results. However, although *Lysobacter* was enriched in N94_A20, it was not included among the final candidate taxa based on correlation analysis. This indicates that differentially enriched taxa do not necessarily correspond directly to changes in functional indices. Their functional significance still needs to be evaluated together with network position, correlation patterns, and further validation [45].

Network analysis showed that both bacterial and fungal communities contained candidate network-relevant OTUs and Zi-Pi topological nodes. Connectors and keystone taxa in co-occurrence networks may help maintain community structure, but network position alone cannot directly prove specific ecological functions [46]. Table 2 further showed that *Holtermanniella* was positively correlated with all five functional indices, and *Naganishia* was positively correlated with the plant nutrient index. In contrast, *Tumebacillus*, *Oxalophagus*, *Variovorax*, *Methylorosula*, and *Talaromyces* were negatively correlated with one or more functional indices. The ecological roles of rhizosphere fungi are affected by host plants, soil environment, and community context [47]. Therefore, these results should be interpreted as associations between candidate taxa and plant–soil functional status, rather than direct causal relationships.

### 4.6. Integrated Interpretation of Plant–Soil–Microbiome Responses to Microbial Inoculation

Taken together, microbial inoculation affected the growth of *P. sylvestris* var. *mongolica* seedlings through coordinated changes in root architecture, plant nutrient accumulation, soil function, and rhizosphere microbial communities, rather than through changes in a single indicator. The co-inoculated treatments, especially N94_A07 and N94_A20, showed different advantages in root architecture, RII, soil AP, soil multifunctionality, and biomass accumulation. Recent studies on synthetic microbial communities and microbial consortia suggest that multi-strain systems can improve plant growth through functional complementarity, community regulation, and rhizosphere resource use, and that their effects usually arise from interactions among multiple members rather than from the function of a single strain [48]. This view is consistent with the treatment- and trait-specific responses observed in this study, involving root architecture, plant nutrition, soil biochemical responses, and rhizosphere microbial community assembly.

Differences among inoculation treatments were also an important finding of this study. N94_A07 mainly showed advantages in root architecture and RII, N94_A20 mainly showed advantages in soil function and biomass accumulation, and N94_A13 showed relatively stable but intermediate responses. Studies on synthetic microbial communities emphasize that strain combination design should not rely only on the growth-promoting ability of individual strains, but should also consider strain compatibility, functional complementarity, rhizosphere colonization ability, and the target plant system [49]. Therefore, the differences among N94_A13, N94_A07, and N94_A20 may reflect different degrees of matching between PGPR strains and N94 in rhizosphere colonization, resource use, and microbial interaction.

It should be noted that the overall interpretation remains based on multiple associated indicators. Although the results on growth, soil properties, plant nutrients, community structure, differential taxa, and network nodes jointly support treatment-specific inoculation responses, correlation analysis and network analysis cannot directly prove the causal roles of key taxa. The stability and predictability of microbiome regulation still require further confirmation through strain functional validation, re-inoculation experiments, and multi-environment testing [50]. Although this study was conducted using *P. sylvestris* var. *mongolica*, the results suggest that fungal–bacterial compatibility may contribute to rhizosphere microecological responses and plant–soil–microbiome coupling in seedling cultivation systems.

### 4.7. Limitations and Future Validation

Although this study analyzed the effects of PGPR-alone, N94-alone, and PGPR–N94 co-inoculation treatments on *Pinus sylvestris* var. *mongolica* seedlings from the perspectives of growth, root traits, plant nutrients, soil function, and the rhizosphere microbiome, several limitations remain. First, the experiment was conducted under greenhouse pot conditions. Although this greenhouse pot experiment allowed controlled comparison among inoculation treatments, the results should not be directly extrapolated to open natural ecosystems because field conditions involve more complex soil heterogeneity, climate variation, and native microbial backgrounds. Therefore, further nursery and field experiments are needed to verify whether the observed inoculation effects can be maintained under natural conditions. In addition, the use of sterilized substrate should be considered when interpreting the rhizosphere microbial results. Sterilization provided a relatively uniform starting background for comparing inoculation treatments, but it also changed the ecological context from modulation of a pre-existing native soil microbiome to recolonization and community assembly in a sterilized growth substrate. Therefore, the microbial community patterns observed in this study should be interpreted as recolonized rhizosphere communities under greenhouse conditions, and their persistence in natural nursery or field soils requires further validation.

Second, candidate key taxa were identified using LEfSe, network topology, and correlation analyses. These approaches are useful for screening potential microbial indicators, but they mainly reveal statistical associations and cannot directly prove causal effects on plant growth or soil function [51]. In addition, the colonization dynamics and interaction processes between N94 and different PGPR strains were not directly examined in this study. Therefore, the observed changes in soil nutrient availability and enzyme activities should be interpreted as responses under greenhouse conditions, and their persistence will depend on the survival, activity, strain compatibility, community stability, and environmental adaptability of the introduced microorganisms [52]. Future studies should combine marker strains, quantitative PCR, co-culture assays, and metabolite profiling to clarify the colonization positions, interaction strength, and resource-use differences among N94, A13, A07, and A20. Further validation under nursery and field conditions is also needed to optimize inoculant formulation, inoculation timing, and inoculation concentration.

## 5. Conclusions

This study showed that PGPR strains, *Suillus luteus* N94, and their co-inoculation treatments improved different aspects of *Pinus sylvestris* var. *mongolica* seedling performance and rhizosphere function. The responses were treatment- and trait-specific rather than uniform across all inoculation types. Among the co-inoculation treatments, N94_A20 was mainly associated with greater seedling height, total dry weight, soil available phosphorus, and soil multifunctionality, whereas N94_A07 was most strongly associated with root length, root surface area, the composite root architecture index, and the relative interaction index. N94_A13 was more closely related to increased ground diameter and phosphorus-related nutrient responses. In addition, single-inoculation treatments also showed specific advantages; for example, N94 contributed to plant nutrient accumulation, and A07 showed a marked plant nutrient response. These findings indicate that fungal–bacterial compatibility determines the dominant plant, soil, and microbial traits improved by co-inoculation. Overall, selected PGPR–N94 combinations have potential for improving conifer seedling cultivation, but the specific functional strengths of both co-inoculation and single-inoculation treatments should be considered when developing microbial inoculants. Further field-based studies are needed to verify the persistence of the introduced strains and their long-term effects on seedling survival, establishment, and rhizosphere microbiome composition under natural conditions.

## Figures and Tables

**Table 1:** Experimental treatments and inoculation design used in this study.

Treatment	Inoculation Type	Biological Component	Inoculum Composition	Role in Comparison
CK	Control	None	50 mL sterile carrier solution	Baseline control
A13	Single inoculation	PGPR strain A13, bacteria	50 mL A13 bacterial suspension	PGPR-alone control
A07	Single inoculation	PGPR strain A07, bacteria	50 mL A07 bacterial suspension	PGPR-alone control
A20	Single inoculation	PGPR strain A20, bacteria	50 mL A20 bacterial suspension	PGPR-alone control
N94	Single inoculation	Fungal strain N94	50 mL N94 fungal inoculum	Fungal-alone control
N94_A13	Combined inoculation	Fungal strain N94 + bacterial PGPR strain A13	25 mL N94 fungal inoculum + 25 mL A13 bacterial suspension	Co-inoculation treatment
N94_A07	Combined inoculation	Fungal strain N94 + bacterial PGPR strain A07	25 mL N94 fungal inoculum + 25 mL A07 bacterial suspension	Co-inoculation treatment
N94_A20	Combined inoculation	Fungal strain N94 + bacterial PGPR strain A20	25 mL N94 fungal inoculum + 25 mL A20 bacterial suspension	Co-inoculation treatment

PGPR, plant growth-promoting rhizobacteria. A13, A07, and A20 represent bacterial PGPR strains. CK, uninoculated control; N94, fungal strain N94. All treatments received a total liquid volume of 50 mL per pot.

**Table 2:** Treatment-level Spearman correlations between candidate microbial taxa and plant–soil functional indices.

Domain	Taxon	Selection Evidence	PGI	RAI	PNI	SNAI	SEAI
Bacteria	*Ramlibacter*	LEfSe differential genus; Top 10 keystone OTU	0.571 *	0.024	0.524	0.119	0.167
	*Massilia*	LEfSe differential genus; Top 10 keystone OTU	0.333	0.524	0.595 *	0.548	0.667 *
	*Serratia*	Top 10 keystone OTU	−0.524	−0.190	−0.286	−0.190	−0.524
	*Pseudarthrobacter*	Top 10 keystone OTU	−0.310	0.262	0.238	0.310	0.143
	*Bacillus*	Top 10 keystone OTU	−0.524	−0.571 *	−0.095	−0.476	−0.476
	*Tumebacillus*	Top 10 keystone OTU	−0.524	−0.810 **	−0.548	−0.833 **	−0.595 *
	*Caenimonas*	Top 10 keystone OTU	−0.048	−0.500	−0.476	−0.643 *	−0.310
	*Oxalophagus*	Top 10 keystone OTU	−0.190	−0.643 *	−0.405	−0.571 *	−0.595 *
	*Variovorax*	Zi-Pi connector	−0.333	−0.595 *	−0.476	−0.714 *	−0.286
	*Methylorosula*	Zi-Pi connector	−0.500	−0.667 *	−0.738 *	−0.833 **	−0.429
Fungi	*Fusarium*	Top 10 keystone OTU; Zi-Pi module hub; LEfSe differential genus	−0.048	−0.095	0.000	0.095	−0.310
	*Tuber*	Top 10 keystone OTU; LEfSe differential genus	−0.167	0.286	0.286	0.310	0.286
	*Tomentella*	Top 10 keystone OTU; LEfSe differential genus	0.143	0.214	−0.190	−0.024	0.119
	*Fusicolla*	Top 10 keystone OTU; LEfSe differential genus	−0.190	−0.500	−0.524	−0.405	−0.452
	*Sphaerosporella*	Top 10 keystone OTU; LEfSe differential genus	0.476	0.143	0.310	0.143	0.429
	*Talaromyces*	Zi-Pi connector; LEfSe differential genus	−0.762 *	−0.381	−0.119	−0.333	−0.405
	*Naganishia*	Zi-Pi connector; LEfSe differential genus	−0.095	0.238	0.643 *	0.452	0.143
	*Holtermanniella*	Zi-Pi connector	0.810 **	0.881 **	0.762 *	0.810 **	0.762 *
	*Mortierella*	LEfSe differential genus	0.548	0.476	0.024	0.429	0.310
	*Clonostachys*	LEfSe differential genus	−0.310	−0.333	−0.524	−0.357	−0.143

Values are Spearman correlation coefficients based on treatment-level means. PGI, plant growth index; RAI, root architecture index; PNI, plant nutrient index; SNAI, soil nutrient availability index; SEAI, soil enzyme activity index. * *p* < 0.05 and ** *p* < 0.01 before FDR correction. Detailed *p* values and FDR-adjusted *p* values are provided in Supplementary Table S4.

## Data Availability

The amplicon sequencing data generated in this study were deposited in the NCBI Sequence Read Archive under BioProject accession number PRJNA730979.

## Supplementary Materials

The following supporting information can be downloaded at: https://www.mdpi.com/article/10.3390/microorganisms14071436/s1, Table S1. Statistical summary of bacterial and fungal diversity and community structure; Table S2. Full LEfSe differential bacterial and fungal genera; Table S3. Keystone OTUs and key Zi-Pi topological nodes in bacterial and fungal networks; Table S4. Full Spearman correlation results between candidate microbial taxa and plant–soil functional indices.

## Abbreviations

The following abbreviations are used in this manuscript:

A07	PGPR strain A07, *Acinetobacter lwoffii* A07
A13	PGPR strain A13, *Serratia plymuthica* A13
A20	PGPR strain A20, *Pseudomonas koreensis* A20
AK	available potassium
AN	available nitrogen
ANOSIM	analysis of similarities
AP	available phosphorus
CFU	colony-forming unit
CK	uninoculated control
DNA	deoxyribonucleic acid
FDR	false discovery rate
ITS	internal transcribed spacer
LDA	linear discriminant analysis
LEfSe	linear discriminant analysis effect size
NB	nutrient broth
NCBI	National Center for Biotechnology Information
N94	fungal strain N94, Suillus luteus N94
N94_A07	co-inoculation of *Suillus luteus* N94 and PGPR strain A07
N94_A13	co-inoculation of *Suillus luteus* N94 and PGPR strain A13
N94_A20	co-inoculation of *Suillus luteus* N94 and PGPR strain A20
NMDS	non-metric multidimensional scaling
OD_600_	optical density at 600 nm
OM	organic matter
OTU	operational taxonomic unit
PCR	polymerase chain reaction
PD	potato dextrose liquid medium
PDA	potato dextrose agar
PERMANOVA	permutational multivariate analysis of variance
PGI	plant growth index
PGPR	plant growth-promoting rhizobacteria
Pi	among-module connectivity
PNI	plant nutrient index
qPCR	quantitative polymerase chain reaction
RAI	root architecture index
RII	relative interaction index
rDNA	ribosomal DNA
rRNA	ribosomal RNA
S-Acp	soil acid phosphatase
S-Cat	soil catalase
S-Sc	soil sucrase
S-Ue	soil urease
SEAI	soil enzyme activity index
SNAI	soil nutrient availability index
SRA	Sequence Read Archive
TK	total potassium
TN	total nitrogen
TP	total phosphorus
Zi	within-module connectivity

## References

[1] Tanaka M., Baek S., Tochigi K., Naganuma T., Inagaki A., Dewi B.S., Koike S. (2023). Conditions affecting ant nesting in stumps in a temperate coniferous planted forest. For. Ecol. Manag..

[2] Yin M.Y., Wu B., Pang Y.J., Wuyun T.N. (2024). Genetic diversity of native provenance and plantation populations of *Pinus sylvestris* var. *mongolica*. Ecol. Evol..

[3] Guo X.Y., Yang G., Ma Y.X., Qiao S., Chen H.Y., Liu F., Ma S. (2025). Effect of plant spacing on the soil properties and fertility of *Pinus sylvestris* var. *mongolica* plantations in sandy land of the agro-pastoral ecotone in northern China. Front. Environ. Sci..

[4] Wen S., Shi Z.J., Zhang X., Pan L.L., Kwon S., Li Y.H., Yang X.H., Li H.Z. (2023). Effect of climate and competition on radial growth of *Pinus sylvestris* var. *mongolica* in Hulunbuir Sandy Land. Plants.

[5] Alzate Zuluaga M.Y., Milani K.M.L., de Souza R.S.C. (2024). Plant–microbe interactions in the rhizosphere for smarter and sustainable crop production. Front. Microbiol..

[6] Khoso M.A., Wagan S., Alam I., Hussain A., Ali Q., Saha S., Poudel T.R., Manghwar H., Liu F. (2024). Impact of plant growth-promoting rhizobacteria (PGPR) on plant nutrition and root characteristics: Current perspective. Plant Stress.

[7] Da Cunha I.C.M., Silva A.V.R., Boleta E.H.M., Pellegrinetti T.A., Zagatto L.F.G., Zagatto S.S.S., Chaves M.G., Mendes R., Patreze C.M., Tsai S.M. (2024). The interplay between the inoculation of plant growth-promoting rhizobacteria and the rhizosphere microbiome and their impact on plant phenotype. Microbiol. Res..

[8] Adedayo A.A., Babalola O.O., Prigent-Combaret C. (2023). Fungi that promote plant growth in the rhizosphere boost crop growth. J. Fungi.

[9] Yun S.N., Hao L.F., Yue Y.J., Liu T.Y., Sun W.N., Shi W.H., Liu Z.Y., Yu J.S., Hu Y.Y. (2026). Regulatory effects of ectomycorrhizal fungi on the morphological construction of *Pinus sylvestris* var. *mongolica* seedlings under exogenous nitrogen input. Trees For. People.

[10] Pandino G., Abbate C., Scavo A., Di Benedetto D., Mauromicale G., Lombardo S. (2024). Co-inoculation of arbuscular mycorrhizal fungi and plant growth promoting bacteria improves plant growth and yield of globe artichoke (*Cynara cardunculus* var. *scolymus*). Sci. Hortic..

[11] Zeng W.L., Xiang D., Li X.M., Gao Q., Chen Y.D., Wang K.M., Qian Y.Y., Wang L.P., Li J., Mi Q.L. (2025). Effects of combined inoculation of arbuscular mycorrhizal fungi and plant growth-promoting rhizosphere bacteria on seedling growth and rhizosphere microecology. Front. Microbiol..

[12] Pang F., Li Q., Solanki M.K., Wang Z., Xing Y.X., Dong D.F. (2024). Soil phosphorus transformation and plant uptake driven by phosphate-solubilizing microorganisms. Front. Microbiol..

[13] Ren Y., Guo M.S., Ding G.D., Wang Y. (2022). Ectomycorrhizal fungi associated with *Pinus sylvestris* var. *mongolica* were altered by soil environments with aging plantation in a semi-arid desert. Front. Environ. Sci..

[14] Liu P., Hu S.Y., Wei H.X., He W.T., Zhou Y.M., Wang Y.T. (2023). Response of radial growth of *Pinus sylvestris* var. *mongolica* of different stand ages to climate and extreme drought events in the semi-arid region of western Liaoning, Northeast China. Front. For. Glob. Change.

[15] Chai Y.N., Futrell S., Schachtman D.P. (2022). Assessment of bacterial inoculant delivery methods for cereal crops. Front. Microbiol..

[16] Armas C., Ordiales R., Pugnaire F.I. (2004). Measuring plant interactions: A new comparative index. Ecology.

[17] Bao S.D. (2000). Soil and Agricultural Chemistry Analysis.

[18] Magoč T., Salzberg S.L. (2011). FLASH: Fast length adjustment of short reads to improve genome assemblies. Bioinformatics.

[19] Edgar R.C. (2013). UPARSE: Highly accurate OTU sequences from microbial amplicon reads. Nat. Methods.

[20] Edgar R.C., Haas B.J., Clemente J.C., Quince C., Knight R. (2011). UCHIME improves sensitivity and speed of chimera detection. Bioinformatics.

[21] Wang Q., Garrity G.M., Tiedje J.M., Cole J.R. (2007). Naive Bayesian classifier for rapid assignment of rRNA sequences into the new bacterial taxonomy. Appl. Environ. Microbiol..

[22] Peterson C.B., Saha S., Do K.M. (2024). Analysis of microbiome data. Annu. Rev. Stat. Appl..

[23] Dixon M.M., Afkairin A., Manter D.K., Vivanco J. (2024). Rhizosphere microbiome co-occurrence network analysis reveals bacterial interactions under low-phosphorus conditions. Microorganisms.

[24] Wickham H., Chang W., Henry L., Pedersen T.L., Takahashi K., Wilke C., Woo K., Yutani H., Dunnington D., van den Brand T. (2026). ggplot2: Create Elegant Data Visualisations Using the Grammar of Graphics. R Package Version 4.0.3. https://CRAN.R-project.org/package=ggplot2.

[25] Ahemad M., Kibret M. (2014). Mechanisms and applications of plant growth promoting rhizobacteria: Current perspective. J. King Saud Univ. Sci..

[26] Rotoni C., Leite M.F., Pijl A., Kowalchuk G.A., Kuramae E.E. (2025). Synergy between AMF and accompanying microbiome enriched with PGPB enhances root development and microbiome dynamics. npj Sustain. Agric..

[27] Qiao Q.H., Wang F.R., Zhang J.X., Chen Y., Zhang C.Y., Liu G.D., Zhang H., Ma C.L., Zhang J. (2017). The variation in the rhizosphere microbiome of cotton with soil type, genotype and developmental stage. Sci. Rep..

[28] Liu F., Hewezi T., Lebeis S.L., Pantalone V., Grewal P.S., Staton M.E. (2019). Soil indigenous microbiome and plant genotypes cooperatively modify soybean rhizosphere microbiome assembly. BMC Microbiol..

[29] Boonlue S., Nacoon S., Ekprasert J., Sanitchon J., Theerakulpisut P., Seemakram P. (2025). The potential of plant growth-promoting fungi enhances the growth, yield, and phytochemical compounds of *Oryza sativa* L. (Maled Phai cultivar) under field conditions. Plants.

[30] Dastogeer K.M.G., Tumpa F.H., Sultana A., Akter M.A., Chakraborty A. (2020). Plant microbiome—An account of the factors that shape community composition and diversity. Curr. Plant Biol..

[31] Lynch J.P. (2019). Root phenotypes for improved nutrient capture: An underexploited opportunity for global agriculture. New Phytol..

[32] Lopez G., Ahmadi S.H., Amelung W., Athmann M., Ewert F., Gaiser T., Gocke M.I., Kautz T., Postma J., Rachmilevitch S. (2023). Nutrient deficiency effects on root architecture and root-to-shoot ratio in arable crops: A meta-analysis. Front. Plant Sci..

[33] Bargaz A., Elhaissoufi W., Khourchi S., Benmrid B., Borden K.A., Rchiad Z. (2021). Benefits of phosphate solubilizing bacteria on belowground crop performance for improved crop acquisition of phosphorus. Microbiol. Res..

[34] Hao L.F., Hao W.Y., Liu T.Y., Zhang M., Xu J.K., Siqinbilige (2021). Effects of ectomycorrhizal fungi inoculation and nitrogen addition on root morphology and nutrient content of *Pinus sylvestris* var. *mongolica* seedlings. J. Beijing For. Univ..

[35] Pan L., Cai B.Y. (2023). Phosphate-solubilizing bacteria: Advances in their physiology, molecular mechanisms and microbial community effects. Microorganisms.

[36] Luo Y., Ma L.G., Feng Q.R., Luo H., Chen C., Wang S.Q., Yuan Y., Liu C., Cao X.L., Li N.N. (2024). Influence and role of fungi, bacteria, and mixed microbial populations on phosphorus acquisition in plants. Agriculture.

[37] Daunoras J., Kačergius A., Gudiukaitė R. (2024). Role of soil microbiota enzymes in soil health and activity changes depending on climate change and the type of soil ecosystem. Biology.

[38] Zhang Y.H., Zhu K.X., Zhuang S.Y., Wang H.L., Cao Z.Z. (2024). Soil nutrient, enzyme activity, and microbial community characteristics of *Eucalyptus urophylla* × *Eucalyptus grandis* plantations in a chronosequence. Forests.

[39] Mehrian S.K., Karimi N., Rahmani F. (2024). Detrimental impacts of concomitant application of cadmium and pesticides are ameliorated by 24-epibrassinolide through alteration in oxidative status and *CYP* genes expression in *Zea mays* L. Rhizosphere.

[40] Chang X.J., Chen J., Zhao K.G., Wang T., Yang Y., Jia X.Y., Hu B.B., Yu Y.M., Li F.X., He Y.H. (2025). Dose-optimized microbial inoculants reshape grape rhizosphere microbiota and enhance fruit quality. Front. Microbiol..

[41] Zhou Y.Y., Liu D.H., Li F.Q., Dong Y.H., Jin Z.L., Liao Y.W.K., Li X.H., Peng S.G., Delgado-Baquerizo M., Li X.G. (2024). Superiority of native soil core microbiomes in supporting plant production. Nat. Commun..

[42] Fu X.H., Huang Y., Fu Q., Qiu Y.B., Zhao J.Y., Li J.X., Wu X.C., Yang Y.H., Liu H.G., Yang X. (2023). Critical transition of soil microbial diversity and composition triggered by plant rhizosphere effects. Front. Plant Sci..

[43] Yue H., Yue W.J., Jiao S., Kim H., Lee Y.H., Wei G.H., Song W.N., Shu D.T. (2023). Plant domestication shapes rhizosphere microbiome assembly and metabolic functions. Microbiome.

[44] Han Q., Zhu G.H., Qiu H.M., Li M.B., Zhang J.M., Wu X.Y., Xiao R.H., Zhang Y., Yang W., Tian B. (2024). Quality traits drive the enrichment of *Massilia* in the rhizosphere to improve soybean oil content. Microbiome.

[45] Baker N.R., Zhalnina K., Yuan M.T., Herman D., Ceja-Navarro J.A., Sasse J., Jordan J.S., Bowen B.P., Wu L.Y., Fossum C. (2024). Nutrient and moisture limitations reveal keystone metabolites linking rhizosphere metabolomes and microbiomes. Proc. Natl. Acad. Sci. USA.

[46] Yang Y.Z., Li Y., Hao K., Zhao Y.J., Li M., Fan Y.J. (2024). Microbial community composition and co-occurrence network analysis of the rhizosphere soil of the main constructive tree species in Helan Mountain of Northwest China. Sci. Rep..

[47] Qiao H.Y., Gao D.L., Yuan T. (2023). Differences in rhizosphere soil fungal communities of wild and cultivated *Paeonia ludlowii* species. Front. Plant Sci..

[48] You T., Liu Q.M., Chen M., Tang S.Y., Ou L.J., Li D.J. (2025). Synthetic microbial communities enhance pepper growth and root morphology by regulating rhizosphere microbial communities. Microorganisms.

[49] Xu X.M., Dinesen C., Pioppi A., Kovács Á.T., Lozano-Andrade C.N. (2025). Composing a microbial symphony: Synthetic communities for promoting plant growth. Trends Microbiol..

[50] Martins S.J., Pasche J., Silva H.A.O., Selten G., Savastano N., Abreu L.M., Bais H.P., Garrett K.A., Kraisitudomsook K., Pieterse C.M.J. (2023). The use of synthetic microbial communities to improve plant health. Phytopathology.

[51] Díaz-Rodríguez A.M., Parra-Cota F.I., Cira-Chávez A.C., García-Ortega L.F., Estrada-Alvarado M.I., Santoyo G., Santos-Villalobos S. (2025). Microbial inoculants in sustainable agriculture: Advancements, challenges, and future directions. Plants.

[52] Zhang Y.X., Jing M.Y., Lyu L.H., Xu X.H., Sun R., Xu X.Y., Chen S.Y., He S.B., Zhang Y.M., Huang P. (2025). Principles for rigorous design and application of synthetic microbial communities. Adv. Sci..

