# Supplementary material for: Strain-Specific Fungal–Bacterial Co-Inoculation Regulates Rhizosphere Microecology and Plant–Soil–Microbiome Responses in Conifer Seedlings
Source: Microorganisms. 2026 Jun 30;14(7):1436. doi: 10.3390/microorganisms14071436 (PMC13414307; doi:10.3390/microorganisms14071436)
Supplement: Supplementary file 1 [file microorganisms-14-01436-s001.zip › microorganisms-4381166-supplementary.pdf]

Supplementary materials

Table 1. Statistical summary of bacterial and fungal diversity and community structure.

Domain	Analysis type	Index or test	Treatment	Mean ± SE / Statistic	Note
Bacteria	Alpha diversity	Chao	CK	2316.9±59.498	
			A13	2431.5±33.759	
			A07	1958.7±86.996	
			A20	2205.7±32.855	
			N94	2626.5±123.94	
			N94_A13	2185.6±121.29	
			N94_A07	2279.2±69.816	
			N94_A20	2434.4±106.25	
		Shannon	CK	5.6085±0.056	
			A13	5.6039±0.011	
			A07	5.2741±0.095	
			A20	5.2093±0.043	
			N94	5.6296±0.0512	
			N94_A13	5.4841±0.0817	
			N94_A07	5.5353±0.1266	
			N94_A20	5.5193±0.0342	
Fungi		Chao	CK	620.67±49.903	
			A13	611.31±33.081	
			A07	606±48.031	
			A20	572.33±10.017	
			N94	882.33±38.991	
			N94_A13	1003.7±52.634	
			N94_A07	927.67±4.042	
			N94_A20	535±4.583	
		Shannon	CK	2.637±0.229	
			A13	3.388±0.078	
			A07	3.5252±0.171	
			A20	3.5182±0.076	
			N94	3.38±0.067	
			N94_A13	3.879±0.046	
			N94_A07	3.7096±0.109	
			N94_A20	2.57±0.363	
Bacteria	Beta diversity	NMDS stress	All treatments	Stress = 0.12	Bray-Curtis distance
		ANOSIM	All treatments	R = 0.8514	999 permutations
Fungi		NMDS stress	All treatments	Stress = 0.119	Bray-Curtis distance
		ANOSIM	All treatments	R = 0.9131	999 permutations

Note: Alpha diversity indices are presented as mean ± SE. Chao was used to estimate community richness. Beta diversity was evaluated based on Bray–Curtis dissimilarity. NMDS stress values indicate ordination reliability, and ANOSIM was used to test differences in community structure among treatments. Bacterial and fungal communities were analyzed separately.

Table 2. Full LEfSe differential bacterial and fungal genera.

Domain	Taxon	Enriched group	Mean abundance (%)	LDA score	P value
Bacteria	*Sphingomonas*	A07	4.56750	4.06945	0.00577
	norank_f__Elev-16S-1332	A07	4.38883	3.80579	0.00630

Domain	Taxon	Enriched group	Mean abundance (%)	LDA score	P value
	Patulibacter	A07	4.23536	3.73588	0.00367
	norank_f__Chitinophagaceae	A07	4.13021	3.63846	0.00410
	Pseudomonas	A07	3.95946	3.58299	0.00397
	Serratia	A13	4.74457	4.40941	0.00243
	Streptomyces	A13	4.27615	3.82512	0.00590
	Rhizomicrobium	A13	4.43976	3.81948	0.01024
	Rhodanobacter	A13	4.10550	3.62534	0.00543
	Pseudarthrobacter	A20	5.09841	4.64097	0.02903
	Bacillus	A20	4.76249	4.22470	0.00537
	Burkholderia-Paraburkholderia	A20	4.61207	4.08389	0.01441
	Phenylobacterium	A20	4.70438	4.06098	0.00717
	Terrabacter	A20	4.29613	3.88060	0.01104
	Pseudolabrys	A20	4.46178	3.84955	0.02821
	Pullulanibacillus	A20	4.17010	3.75301	0.00312
	Dyella	A20	4.01580	3.64980	0.00240
	Tumebacillus	CK	4.61138	4.19659	0.00299
	Candidatus_Solibacter	CK	4.38059	3.88031	0.00379
	Caenimonas	CK	4.29045	3.80626	0.00369
	Mesorhizobium	CK	4.27969	3.69067	0.01412
	norank_o__Acidimicrobiales	CK	4.12500	3.60274	0.00302
	Variovorax	CK	3.93843	3.53837	0.00577
	Oxalophagus	CK	4.01333	3.53373	0.00513
	Ramlibacter	N94	4.83036	4.12145	0.01514
	norank_c__Acidobacteria	N94	4.42378	3.95223	0.02393
	Bryobacter	N94	4.21879	3.66352	0.00427
	norank_f__Nitrosomonadaceae	N94	4.12076	3.62775	0.00677
	Flavisolibacter	N94	4.23323	3.58518	0.01819
	norank_f__AKYH478	N94_A07	3.96758	3.54127	0.00253
	Nocardioides	N94_A13	4.62418	4.17022	0.03449
	Gemmatimonas	N94_A13	4.79407	4.16630	0.01008
	Devosia	N94_A13	4.23846	3.54302	0.01077
	Massilia	N94_A20	4.74557	4.14291	0.00465
	norank_p__Saccharibacteria	N94_A20	4.51573	3.99229	0.00408
	norank_f__ABS-19	N94_A20	4.05880	3.70152	0.00315
	Lysobacter	N94_A20	4.15100	3.62521	0.00857
	Blastococcus	N94_A20	3.95972	3.50356	0.01723
	Paraphaeosphaeria	A07	4.72135	4.18382	0.02139
	Cryptococcus_f__Tremellaceae	A07	3.92184	3.96810	0.00447
	Nematoctonus	A07	3.26605	3.81759	0.02710
	Mucor	A07	2.77772	3.77723	0.01274
	Naganishia	A07	3.97653	3.73000	0.00893
	Dipodascus	A07	2.56300	3.66742	0.04607
	Rhodotorula	A07	3.79072	3.53374	0.00923
Fungi	*Solicoccozyma*	A07	3.58165	3.52135	0.00577
	Knufia	A07	3.04138	3.51026	0.00404
	Tomentella	A13	5.48694	5.10642	0.00281
	Zopfiella	A13	4.18547	3.90295	0.00314
	Penicillium	A20	5.40099	5.02197	0.00339
	Talaromyces	A20	5.35407	4.96390	0.00265
	Tuber	A20	5.12271	4.77167	0.00378
	Trichoderma	A20	4.86849	4.50402	0.00888

Domain	Taxon	Enriched group	Mean abundance (%)	LDA score	P value
	Chaetomium	A20	3.80741	3.50303	0.00790
	Fusicolla	CK	5.28414	4.90601	0.00343
	Aspergillus	N94	4.47343	4.13253	0.00432
	Wallemia	N94	2.78167	3.75097	0.01426
	Phialemonium	N94	3.91440	3.65072	0.00309
	Humicola	N94_A07	4.53960	4.24812	0.01892
	Clonostachys	N94_A07	4.66486	4.20012	0.00385
	Plenodomus	N94_A07	2.80203	3.84893	0.00277
	Conlarium	N94_A07	2.46571	3.70172	0.02401
	Latorua	N94_A07	2.79635	3.66391	0.01753
	Fusarium	N94_A13	5.66509	5.28918	0.00408
	Golubevia	N94_A13	2.34862	4.05298	0.02387
	Schizothecium	N94_A13	2.58537	3.95381	0.01414
	Sarocladium	N94_A13	3.07275	3.58169	0.01213
	Plectosphaerella	N94_A13	3.65999	3.56015	0.00921
	Sphaerosporella	N94_A20	5.77931	5.43061	0.00232
	Mortierella	N94_A20	4.73594	4.36093	0.00973
	Cladosporium	N94_A20	4.67899	4.33388	0.00862
	Peziza	N94_A20	4.10577	3.89096	0.01516
	Acremonium	N94_A20	3.71309	3.58554	0.00363
	Ampelomyces	N94_A20	3.16848	3.56124	0.04519

Table 3. Keystone OTUs and key Zi-Pi topological nodes in bacterial and fungal networks.

Domain	Evidence type	Node/OTU ID	Taxon / Genus	Ks value	Zi-Pi role	Note
Bacteria	Top 10 keystone OTU	OTU826	*Cyanobacteria*	0.208		Bacterial keystone OTU ranked by structural keystoneness (Ks); genus annotation not available in current OTU table.
		OTU708	*Serratia*	0.122		Bacterial keystone OTU ranked by Ks; genus annotation not available in current OTU table.
		OTU3726	*Pseudarthrobacter*	0.048		Bacterial keystone OTU ranked by Ks; genus annotation not available in current OTU table.
		OTU2806	*Bacillus*	0.019		Bacterial keystone OTU ranked by Ks; genus annotation not available in current OTU table.
		OTU126	*Ramlibacter*	0.016		Bacterial keystone OTU ranked by Ks; genus annotation not

Domain	Evidence type	Node/OTU ID	Taxon / Genus	Ks value	Zi-Pi role	Note
						available in current OTU table.
		OTU14	*Tumebacillus*	0.016		Bacterial keystone OTU ranked by Ks; genus annotation not available in current OTU table.
		OTU2218	*Caenimonas*	0.015		Bacterial keystone OTU ranked by Ks; genus annotation not available in current OTU table.
		OTU5324	*Massilia*	0.014		Bacterial keystone OTU ranked by Ks; genus annotation not available in current OTU table.
		OTU1907	*Oxalophagus*	0.013		Bacterial keystone OTU ranked by Ks; genus annotation not available in current OTU table.
		OTU2531	*Ramlibacter*	0.013		Bacterial keystone OTU ranked by Ks; genus annotation not available in current OTU table.
	Zi-Pi key node		*Variovorax*		Connector	Genus-level connector node in bacterial Zi-Pi analysis.
			Methylorosula		Connector	Genus-level connector node in bacterial Zi-Pi analysis.
Fungi	Top 10 keystone OTU	OTU1175	*unclassified_k__Fungi*	0.301		Fungal keystone OTU ranked by structural keystoneness (Ks).
		OTU603	*Tuber*	0.222		Fungal keystone OTU ranked by Ks.
		OTU6172	*Tomentella*	0.150		Fungal keystone OTU ranked by Ks.
		OTU6319	*unclassified_p__Ascomycota*	0.108		Fungal keystone OTU ranked by Ks.
		OTU3474	*Fusarium*	0.094		Fungal keystone OTU ranked by Ks.
		OTU358	*Fusicolla*	0.088		Fungal keystone OTU ranked by Ks.
		OTU4908	*Tomentella*	0.084		Fungal keystone OTU ranked by Ks.
		OTU3261	*Fusarium*	0.082		Fungal keystone OTU

Domain	Evidence type	Node/OTU ID	Taxon / Genus	Ks value	Zi-Pi role	Note
						ranked by Ks.
		OTU1466	*Sphaerosporella*	0.082		Fungal keystone OTU ranked by Ks.
		OTU5330	*Fusarium*	0.081		Fungal keystone OTU ranked by Ks.
	Zi-Pi key node		*Talaromyces*		Connector	Genus-level connector node in fungal Zi-Pi analysis.
			Naganishia		Connector	Genus-level connector node in fungal Zi-Pi analysis.
			Holtermanniella		Connector	Genus-level connector node in fungal Zi-Pi analysis.
			Fusarium		Module hub	Genus-level module hub in fungal Zi-Pi analysis.

Note: This simplified table lists only the top keystone OTUs and the key Zi-Pi topological nodes (connectors and module hubs) used to support Figure 6. Ordinary peripheral nodes are not shown. Ks, structural keystoneness; Zi-Pi role, topological role identified by Zi-Pi analysis.

Table 4. Full Spearman correlation results between candidate microbial taxa and plant–soil functional indices.

Domain	Taxon	Functional index	Spearman rho	P value	FDR-adjusted P value	Significance
Bacteria	*Ramlibacter*	Plant growth index	0.5710	0.1390	0.5374	ns
Bacteria	*Ramlibacter*	Root architecture index	0.0240	0.9554	0.9651	ns
Bacteria	*Ramlibacter*	Plant nutrient index	0.5240	0.1827	0.5374	ns
Bacteria	*Ramlibacter*	Soil nutrient availability index	0.1190	0.7789	0.8654	ns
Bacteria	*Ramlibacter*	Soil enzyme activity index	0.1670	0.6932	0.8457	ns
Bacteria	*Massilia*	Plant growth index	0.3330	0.4198	0.7009	ns
Bacteria	*Massilia*	Root architecture index	0.5240	0.1827	0.5374	ns
Bacteria	*Massilia*	Plant nutrient index	0.5950	0.1195	0.5374	ns
Bacteria	*Massilia*	Soil nutrient availability index	0.5480	0.1600	0.5374	ns

Domain	Taxon	Functional index	Spearman rho	P value	FDR-adjusted P value	Significance
Bacteria	*Massilia*	Soil enzyme activity index	0.6670	0.0710	0.5350	ns
Bacteria	*Serratia*	Plant growth index	-0.5240	0.1827	0.5374	ns
Bacteria	*Serratia*	Root architecture index	-0.1900	0.6514	0.8246	ns
Bacteria	*Serratia*	Plant nutrient index	-0.2860	0.4927	0.7039	ns
Bacteria	*Serratia*	Soil nutrient availability index	-0.1900	0.6514	0.8246	ns
Bacteria	*Serratia*	Soil enzyme activity index	-0.5240	0.1827	0.5374	ns
Bacteria	*Pseudarthrobacter*	Plant growth index	-0.3100	0.4556	0.7009	ns
Bacteria	*Pseudarthrobacter*	Root architecture index	0.2620	0.5309	0.7477	ns
Bacteria	*Pseudarthrobacter*	Plant nutrient index	0.2380	0.5702	0.7811	ns
Bacteria	*Pseudarthrobacter*	Soil nutrient availability index	0.3100	0.4556	0.7009	ns
Bacteria	*Pseudarthrobacter*	Soil enzyme activity index	0.1430	0.7358	0.8457	ns
Bacteria	*Bacillus*	Plant growth index	-0.5240	0.1827	0.5374	ns
Bacteria	*Bacillus*	Root architecture index	-0.5710	0.1390	0.5374	ns
Bacteria	*Bacillus*	Plant nutrient index	-0.0950	0.8225	0.8750	ns
Bacteria	*Bacillus*	Soil nutrient availability index	-0.4760	0.2329	0.5416	ns
Bacteria	*Bacillus*	Soil enzyme activity index	-0.4760	0.2329	0.5416	ns
Bacteria	*Tumebacillus*	Plant growth index	-0.5240	0.1827	0.5374	ns
Bacteria	*Tumebacillus*	Root architecture index	-0.8100	0.0149	0.2483	*
Bacteria	*Tumebacillus*	Plant nutrient index	-0.5480	0.1600	0.5374	ns
Bacteria	*Tumebacillus*	Soil nutrient availability	-0.8330	0.0102	0.2483	*

Domain	Taxon	Functional index	Spearman rho	P value	FDR-adjusted P value	Significance
		index				
Bacteria	*Tumebacillus*	Soil enzyme activity index	-0.5950	0.1195	0.5374	ns
Bacteria	*Caenimonas*	Plant growth index	-0.0480	0.9108	0.9488	ns
Bacteria	*Caenimonas*	Root architecture index	-0.5000	0.2070	0.5416	ns
Bacteria	*Caenimonas*	Plant nutrient index	-0.4760	0.2329	0.5416	ns
Bacteria	*Caenimonas*	Soil nutrient availability index	-0.6430	0.0856	0.5350	ns
Bacteria	*Caenimonas*	Soil enzyme activity index	-0.3100	0.4556	0.7009	ns
Bacteria	*Oxalophagus*	Plant growth index	-0.1900	0.6514	0.8246	ns
Bacteria	*Oxalophagus*	Root architecture index	-0.6430	0.0856	0.5350	ns
Bacteria	*Oxalophagus*	Plant nutrient index	-0.4050	0.3199	0.6273	ns
Bacteria	*Oxalophagus*	Soil nutrient availability index	-0.5710	0.1390	0.5374	ns
Bacteria	*Oxalophagus*	Soil enzyme activity index	-0.5950	0.1195	0.5374	ns
Bacteria	*Variovorax*	Plant growth index	-0.3330	0.4198	0.7009	ns
Bacteria	*Variovorax*	Root architecture index	-0.5950	0.1195	0.5374	ns
Bacteria	*Variovorax*	Plant nutrient index	-0.4760	0.2329	0.5416	ns
Bacteria	*Variovorax*	Soil nutrient availability index	-0.7140	0.0465	0.4227	*
Bacteria	*Variovorax*	Soil enzyme activity index	-0.2860	0.4927	0.7039	ns
Bacteria	*Methylorosula*	Plant growth index	-0.5000	0.2070	0.5416	ns
Bacteria	*Methylorosula*	Root architecture index	-0.6670	0.0710	0.5350	ns
Bacteria	*Methylorosula*	Plant nutrient index	-0.7380	0.0366	0.3660	*
Bacteria	*Methylorosul*	Soil nutrient	-0.8330	0.0102	0.2483	*

Domain	Taxon	Functional index	Spearman rho	P value	FDR-adjusted P value	Significance
	a	availability index				
Bacteria	*Methylorosula*	Soil enzyme activity index	-0.4290	0.2894	0.6029	ns
Fungi	*Fusarium*	Plant growth index	-0.0480	0.9108	0.9488	ns
Fungi	*Fusarium*	Root architecture index	-0.0950	0.8225	0.8750	ns
Fungi	*Fusarium*	Plant nutrient index	0.0000	1.0000	1.0000	ns
Fungi	*Fusarium*	Soil nutrient availability index	0.0950	0.8225	0.8750	ns
Fungi	*Fusarium*	Soil enzyme activity index	-0.3100	0.4556	0.7009	ns
Fungi	*Tuber*	Plant growth index	-0.1670	0.6932	0.8457	ns
Fungi	*Tuber*	Root architecture index	0.2860	0.4927	0.7039	ns
Fungi	*Tuber*	Plant nutrient index	0.2860	0.4927	0.7039	ns
Fungi	*Tuber*	Soil nutrient availability index	0.3100	0.4556	0.7009	ns
Fungi	*Tuber*	Soil enzyme activity index	0.2860	0.4927	0.7039	ns
Fungi	*Tomentella*	Plant growth index	0.1430	0.7358	0.8457	ns
Fungi	*Tomentella*	Root architecture index	0.2140	0.6103	0.8246	ns
Fungi	*Tomentella*	Plant nutrient index	-0.1900	0.6514	0.8246	ns
Fungi	*Tomentella*	Soil nutrient availability index	-0.0240	0.9554	0.9651	ns
Fungi	*Tomentella*	Soil enzyme activity index	0.1190	0.7789	0.8654	ns
Fungi	*Fusicolla*	Plant growth index	-0.1900	0.6514	0.8246	ns
Fungi	*Fusicolla*	Root architecture index	-0.5000	0.2070	0.5416	ns
Fungi	*Fusicolla*	Plant nutrient index	-0.5240	0.1827	0.5374	ns

Domain	Taxon	Functional index	Spearman rho	P value	FDR-adjusted P value	Significance
Fungi	*Fusicolla*	Soil nutrient availability index	-0.4050	0.3199	0.6273	ns
Fungi	*Fusicolla*	Soil enzyme activity index	-0.4520	0.2604	0.5787	ns
Fungi	*Sphaerosporella*	Plant growth index	0.4760	0.2329	0.5416	ns
Fungi	*Sphaerosporella*	Root architecture index	0.1430	0.7358	0.8457	ns
Fungi	*Sphaerosporella*	Plant nutrient index	0.3100	0.4556	0.7009	ns
Fungi	*Sphaerosporella*	Soil nutrient availability index	0.1430	0.7358	0.8457	ns
Fungi	*Sphaerosporella*	Soil enzyme activity index	0.4290	0.2894	0.6029	ns
Fungi	*Talaromyces*	Plant growth index	-0.7620	0.0280	0.3111	*
Fungi	*Talaromyces*	Root architecture index	-0.3810	0.3518	0.6765	ns
Fungi	*Talaromyces*	Plant nutrient index	-0.1190	0.7789	0.8654	ns
Fungi	*Talaromyces*	Soil nutrient availability index	-0.3330	0.4198	0.7009	ns
Fungi	*Talaromyces*	Soil enzyme activity index	-0.4050	0.3199	0.6273	ns
Fungi	*Naganishia*	Plant growth index	-0.0950	0.8225	0.8750	ns
Fungi	*Naganishia*	Root architecture index	0.2380	0.5702	0.7811	ns
Fungi	*Naganishia*	Plant nutrient index	0.6430	0.0856	0.5350	ns
Fungi	*Naganishia*	Soil nutrient availability index	0.4520	0.2604	0.5787	ns
Fungi	*Naganishia*	Soil enzyme activity index	0.1430	0.7358	0.8457	ns
Fungi	*Holtermanniella*	Plant growth index	0.8100	0.0149	0.2483	*
Fungi	*Holtermanniella*	Root architecture index	0.8810	0.0039	0.2483	**
Fungi	*Holtermanniella*	Plant nutrient	0.7620	0.0280	0.3111	*

Domain	Taxon	Functional index	Spearman rho	P value	FDR-adjusted P value	Significance
		index				
Fungi	*Holtermanniella*	Soil nutrient availability index	0.8100	0.0149	0.2483	*
Fungi	*Holtermanniella*	Soil enzyme activity index	0.7620	0.0280	0.3111	*
Fungi	*Mortierella*	Plant growth index	0.5480	0.1600	0.5374	ns
Fungi	*Mortierella*	Root architecture index	0.4760	0.2329	0.5416	ns
Fungi	*Mortierella*	Plant nutrient index	0.0240	0.9554	0.9651	ns
Fungi	*Mortierella*	Soil nutrient availability index	0.4290	0.2894	0.6029	ns

Note: Spearman correlations were calculated based on treatment-level means. Candidate microbial taxa were selected according to LEfSe differential abundance, structural keystoneness, and/or Zi-Pi topological roles. FDR-adjusted P values were calculated using the Benjamini–Hochberg method. *, $P < 0.05$; **, $P < 0.01$; ***, $P < 0.001$ before FDR correction; ns, not significant.
